# The demographic and geographic impact of the COVID pandemic in Bulgaria and Eastern Europe in 2020

**DOI:** 10.1038/s41598-022-09790-w

**Published:** 2022-04-15

**Authors:** Antoni Rangachev, Georgi K. Marinov, Mladen Mladenov

**Affiliations:** 1 Department of Mathematics, University of Chicago, Chicago, IL 60637 USA; 2 Institute of Mathematics and Informatics, Bulgarian Academy of Sciences, Sofia, 1113 Bulgaria; 3 Department of Genetics, Stanford University, Stanford, CA 94305 USA; 4 Premier Research, Morrisville, NC 27560 USA

**Keywords:** Diseases, Health care, Risk factors

## Abstract

The COVID-19 pandemic followed a unique trajectory in Eastern Europe compared to other heavily affected regions, with most countries there only experiencing a major surge of cases and deaths towards the end of 2020 after a relatively uneventful first half of the year. However, the consequences of that surge have not received as much attention as the situation in Western countries. Bulgaria, even though it has been one of the most heavily affected countries, has been one of those neglected cases. We use mortality and mobility data from Eurostat, official governmental and other sources to examine the development and impact of the COVID-19 pandemic in Bulgaria and other European countries. We find a very high level of excess mortality in Eastern European countries measured by several metrics including excess mortality rate (EMR), P-scores, potential years of life lost (PYLL) and its age standardised version (ASYR), and working years of life lost (WYLL). By the last three metrics Eastern Europe emerges as the hardest hit region by the pandemic in Europe in 2020. With a record EMR at ~0.27% and a strikingly large and mostly unique to it mortality rate in the working age (15–64 years) demographics, Bulgaria emerges as one of the most affected countries in Eastern Europe. The high excess mortality in Bulgaria correlates with insufficient intensity of testing, with delayed imposition of “lockdown” measures, and with high prevalence of cardiovascular diseases. We also find major geographic and demographic disparities within the country, with considerably lower mortality observed in major cities relative to more remote areas (likely due to disparities in the availability of medical resources). Analysis of the course of the epidemic revealed that individual mobility measures were predictive of the eventual decline in cases and deaths. However, while mobility declined as a result of the imposition of a lockdown, it already trended downwards before such measures were introduced, which resulted in a reduction of deaths independent of the effect of restrictions. Large excess mortality and high numbers of potential years of life lost are observed as a result of the COVID pandemic in Bulgaria, as well as in several other countries in Eastern Europe. Significant delays in the imposition of stringent mobility-reducing measures combined with a lack of medical resources likely caused a substantial loss of life, including in the working age population.

## Introduction

The SARS-CoV-2 virus and COVID-19, the disease it causes^1–3^, have emerged as the most acute public health emergency in a century. The novel coronavirus spread rapidly before significant efforts at containment were implemented in much of the world, resulting in devastating early outbreaks in the United States and Western Europe, starting in late February and early March of 2020.

Some combination of lockdown measures, imposed in response to surging infections, voluntary changes in behavior, and the onset of the summer season is thought to have caused the major decline in COVID-19 cases in Europe in the summer of 2020. However, winter in the Southern hemisphere, during which large epidemics developed in South Africa and South America, together with the well-documented seasonality of common-cold coronaviruses^4^, strongly suggested that a major second wave was to be expected in Europe with the arrival of winter^5^, and when it eventually arrived expectation turned into reality.

During the early months of the pandemic, a dichotomy emerged between countries in Western and Eastern Europe (with the possible exception of Russia). Western Europe was heavily affected—by June 2020 official COVID mortality reached 600 to 800 deaths per million (DPM) in countries such as Spain, Italy, the UK, Belgium, France, and Sweden, with excess mortality rates even higher^6–10^. In contrast, most Eastern European countries registered relatively few deaths, possibly because of much earlier implementation of social distancing measures relative to the development of the outbreak.

This dichotomy disappeared during the second wave at the end of 2020, with both countries in Western and Eastern Europe officially registering a large number of COVID-related fatalities, as well as in some cases considerably larger excess mortality. However, the development of the pandemic in Eastern Europe has so far generally received much less attention than that in the West even though multiple countries in the region were heavily affected by it. We show this using multiple excess mortality measures, which quantify the pandemic-related loss of life and allow for standardized comparisons between countries.

Among Eastern European countries, Bulgaria has emerged as perhaps the most heavily affected by the pandemic as suggested by excess mortality analysis^6^. Here we analyze the development and impact of the pandemic on Bulgaria, in the broader European context, across demographic groups within the country, and for its regional subdivisions, as well as the influence of human mobility changes and government-imposed quarantine measure on the course of the pandemic. We use these analyses to identify correlate factors likely responsible for particularly high unexplained excess mortality in certain settings.

**Figure 1:**
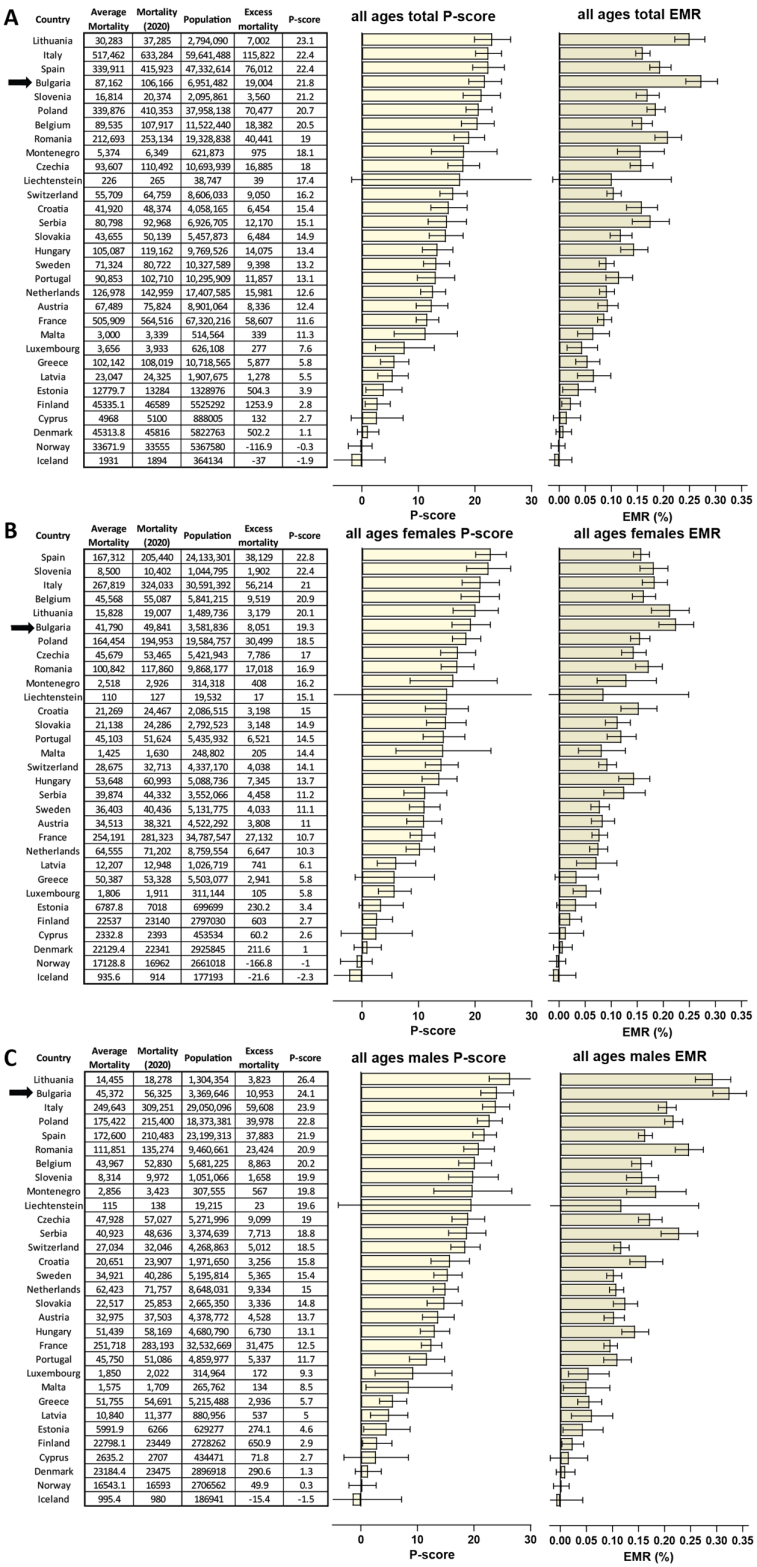
Excess mortality in Bulgaria and other European countries in 2020. (**A**) Overall P-scores and excess mortality (in deaths per million; DPM) for all ages in Bulgaria (highlighted in red) and other European countries; (**B**) P-scores and excess mortality for females of all ages; (**C**) P-scores and excess mortality for males of all ages. All error bars in this and subsequent figures represent 95% confidence intervals.

**Figure 2:**
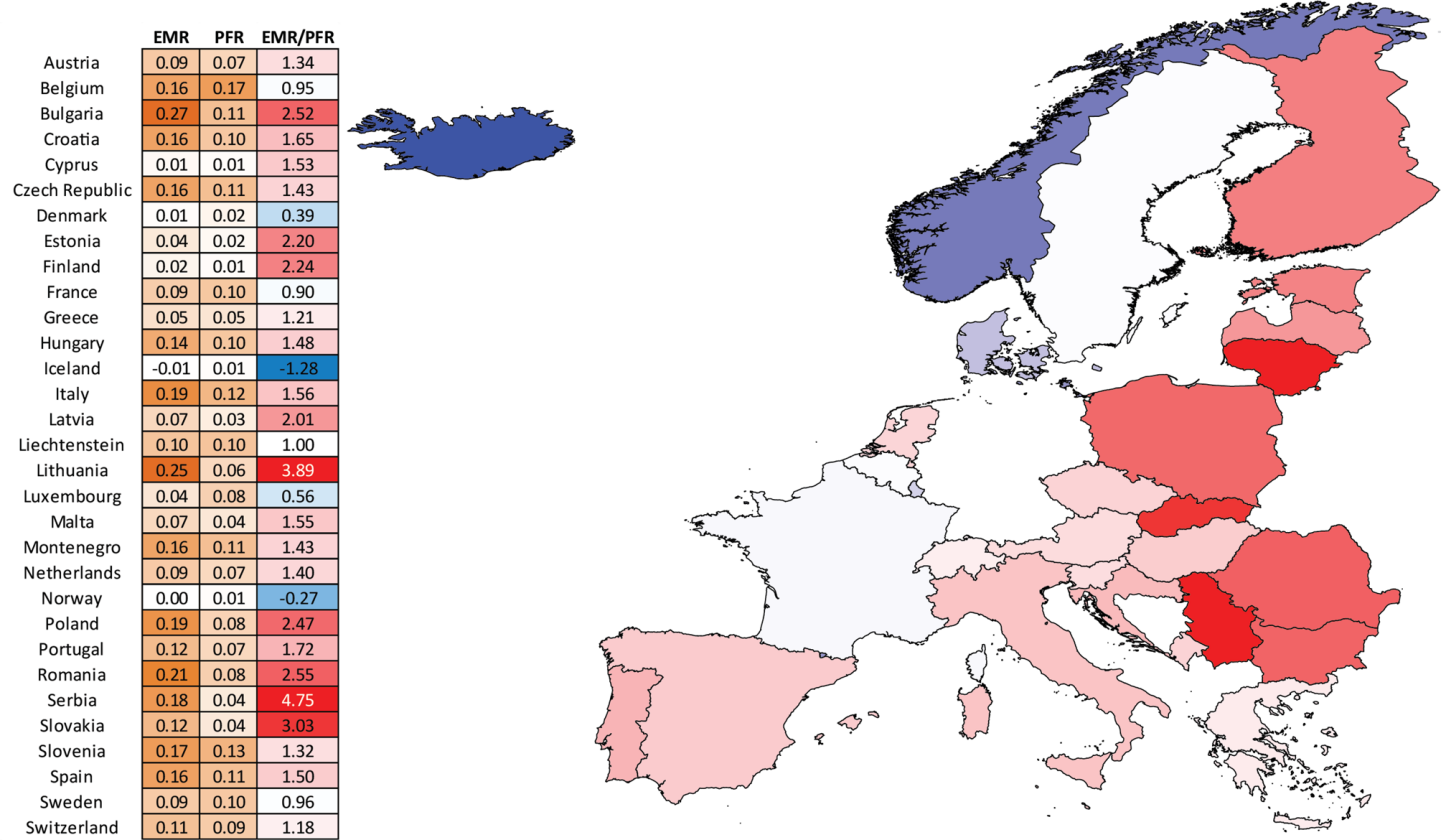
Ratio between excess mortality and official COVID-attributed deaths in European countries in 2020. Note that the high EMR/PFR ratios for 2020 in countries like Finland and Estonia might be an artifact of overall low both excess and COVID-attributed mortality.

**Figure 3:**
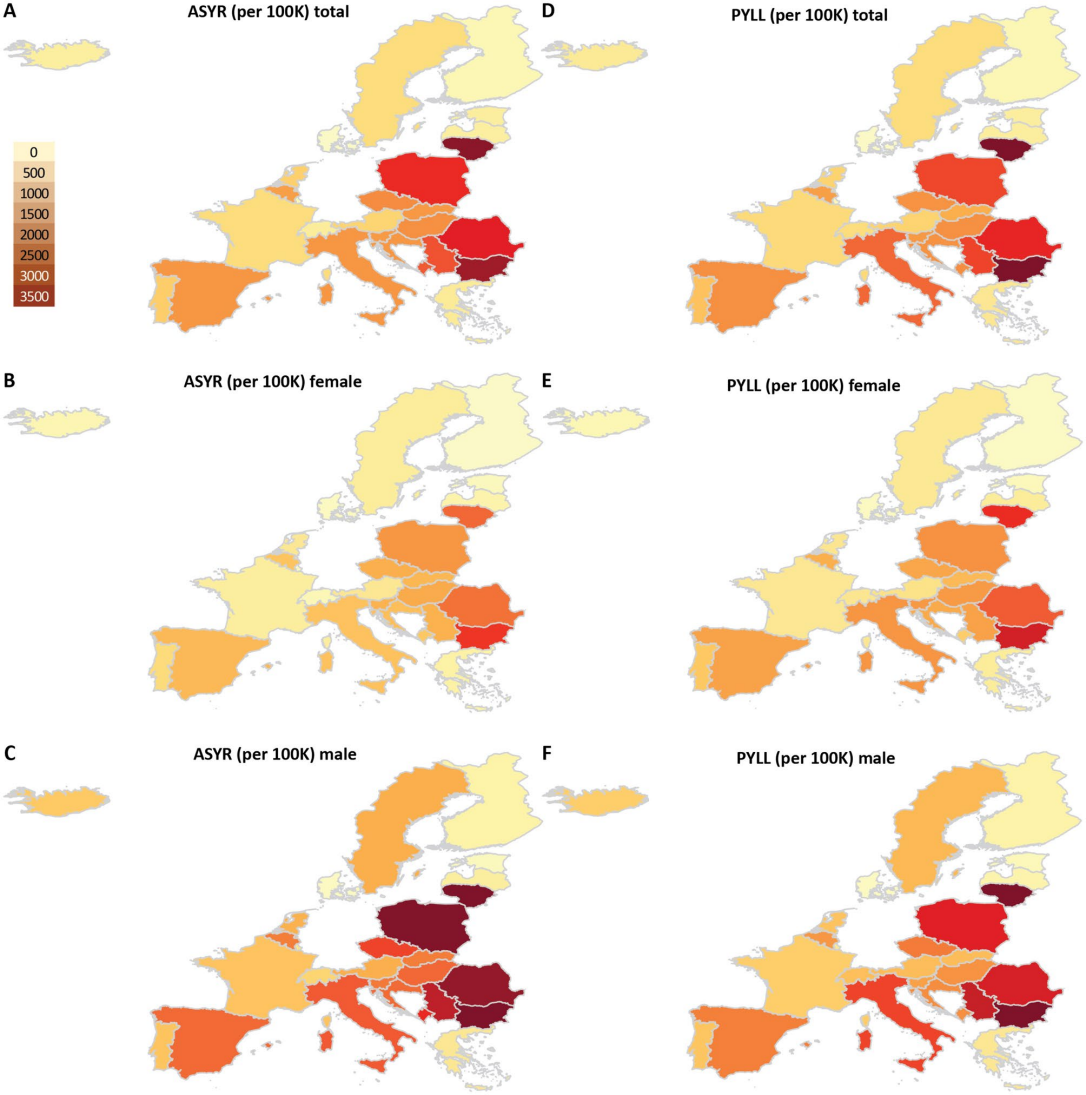
Geographic distribution of excess mortality-based ASYR and PYLL values for European countries in 2020. Shown are the total (per 100K people) values. (**A**) ASYR values for the whole population; (**B**) ASYR values for females; (**C**) ASYR values for males; (**D**) PYLL values for the whole population; (**E**) PYLL values for females; (**F**) PYLL values for males.

**Figure 4:**
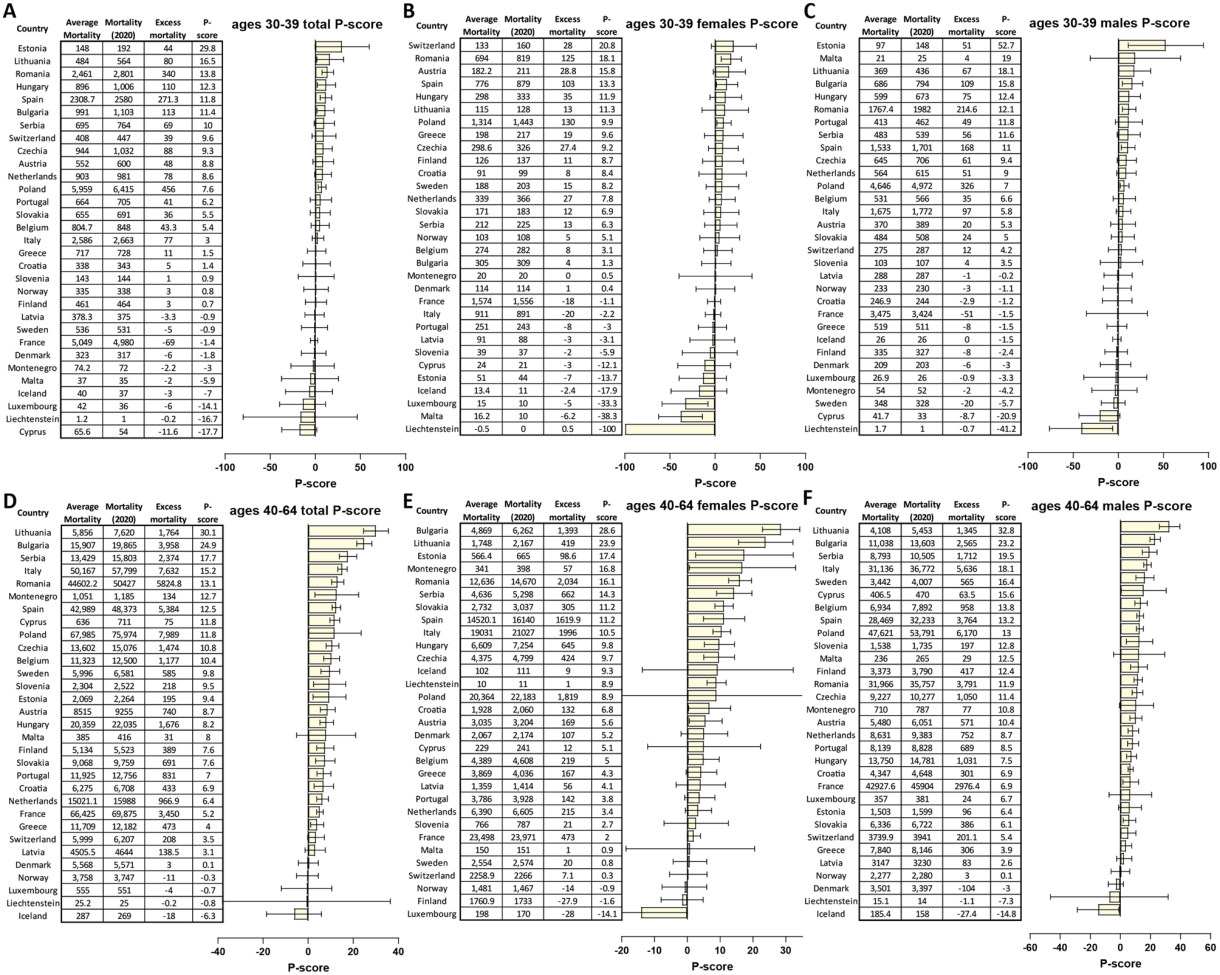
Excess mortality in working age populations in Bulgaria and other European countries in 2020. (**A**) P-scores for the overall population in ages 30–39; (**B**) P-scores for females in ages 30–39; (**C**) P-scores for males in ages 30–39; (**D**) P-scores for the overall population in ages 40–64; (**E**) P-scores for females in ages 40–64; (**F**) P-scores for males in ages 40–64.

## Results

### Mortality during the COVID pandemic in Bulgaria

We analyzed overall excess mortality patterns in Bulgaria for the year 2020 and compared it to data for other European countries, which submit mortality data to Eurostat, for the same period. We focus on excess mortality rather than officially registered COVID deaths because limited testing and varying standards for official reporting of COVID deaths can result in large disparities between public figures for COVID-related mortality and the actual burden the disease has imposed on the population^6^. While some of the excess deaths are caused by the collapse of healthcare services during peak moments of COVID waves, when a particularly large discrepancy between official COVID deaths and excess deaths is observed, and when the shapes of the excess mortality and official COVID case and death counts match closely, excess mortality is likely mostly due to underreporting of COVID deaths due to insufficient testing and other irregularities like reporting almost exclusively COVID-19 deaths that occurred in hospitals as it is the case with Bulgaria.

In total, we estimate that 19,004 lives have been lost in Bulgaria in 2020 in excess of the baseline from previous years (Fig. 1A). This amounts to an EMR of 2,734 DPM, or ~0.27%, for the year and ranks the country as the most highly affected within the EU (Fig. 1A; according to P-scores Lithuania, Italy and Spain rank higher). COVID mortality is in most countries higher in males than in females^32^, and this is also what is observed in Bulgaria and most other EU countries (Fig. 1B,C). For females, an EMR of 2,248 DPM is observed (P-score of 19.3), compared to an EMR of 3,250 DPM for males (P-score of 24.1) across all ages.

These observed EMR values are much higher than the officially reported COVID-attributed population fatality rate (PFR), by a factor of ~2.5$\times$. Examination of the EMR/PFR ratios in Europe showed that excess deaths are higher than official COVID death tolls in most countries (Fig. 2). However, a clear dichotomy emerges between Eastern and Western Europe, with the EMR/PFR ratio being considerably higher in countries in Eastern Europe such as Bulgaria, Romania, Poland, Slovakia, Lithuania, and others.

These estimates and geographic patterns are in agreement with other recent analyses of excess mortality^33,34^.

### Loss of life as a result of the COVID pandemic

We next examined the impact of the pandemic in terms of years of life lost using the PYLL and ASYR metrics based on excess mortality (Fig. 3), where the latter metric is the more suitable one for cross-country comparison. Both metrics paint a similar picture. However, there are some notable differences when using P-scores from (Fig. 1). For instance, Italy, Spain and Belgium are among the countries with highest P-scores - 2nd, 3rd, 7th, respectively – but in terms of ASYR valus these countries rank 9th, 10th, and 14th.

Using standardized ASYR and PYLL values (per 100,000 population; Supplementary Figs. 2A-C and 1), we find that the highest total loss of life among the examined countries occurred in Lithuania and Bulgaria for both males and females, followed by Romania, Poland, Serbia, Montenegro, Czechia and Hungary. Out of the top 13 countries in Supplementary Fig. 1A 11 are in Eastern Europe. This higher loss of life burden in Eastern European countries is explained not only by their high EMRs but also by a large numbers of deaths in younger age groups ($<65$ years). Lithuania and Bulgaria exhibit 3, 351 and 3, 195 years of life lost per 100K standardised population, whereas countries with similar P-scores like Italy and Spain show 1, 506 and 1, 498 years of life lost per 100K standardised population, respectively. This drastic difference is explained by the age distribution of excess deaths relative to the life expectancy for each country.

Calculation of WYLL values, which show the loss of working years of life, showed Lithuania and Bulgaria to have incurred the highest such loss within the set of examined countries (Supplementary Fig. 2D-E; note that the high total WYLL value for Iceland is possibly an artifact of the small population of the country). In Lithuania and Bulgaria, $25\%$ and $21\%$ of excess deaths, respectively, are of people in the age group 40–64. In contrast, only $7\%$ of excess deaths in Italy and Spain are in the age group 40–64 (see Fig. 4D).

In countries such as Italy, Spain, France and Belgium, only 17–20% of excess deaths are under 75 years of age, while 67–70% of all excess deaths in these countries are in the age group $80+$.

For Bulgaria, we find an average PYLL value of $12.91 \pm 0.08$ in total, $12.24 \pm 0.24$ for males, and $12.67 \pm 0.08$ per female (Supplementary Fig. 2). Excluding outliers (note that average PYLL values based on excess mortality are very high in countries such as Iceland, Luxembourg due to stochasticity associated with the very low number of excess deaths), these values are generally higher than what is seen in Western Europe. The only three countries with an average PYLL $\ge$13 are Estonia, Lithuania, Serbia, and Bulgaria, compared to values in the 7 to 9.5 years range for countries such as Switzerland, Sweden and Belgium. Despite males exhibiting higher mortality due to COVID-19, the average PYLL based on excess deaths in Bulgaria is higher for females (it is also higher for females in several other European countries; Supplementary Fig. 3).

Using official COVID-attributed deaths, for Bulgaria we obtain an average of 12.37 years lost for males and 14.01 years lost for females. Based on the official COVID-19 mortality data for Czechia (the other country for which exact data about the age of the diseased was available to us) we obtain 9.78 and 9.35 for males and females, respectively. In both cases, the estimates we obtain for the average PYLLs from excess mortality and official COVID-19 deaths data are in agreement (note, however, that there are substantial differences between Bulgaria and Czechia in other aspects – for example, the average age of officially registered COVID-19 deaths for women is 71 years in Bulgaria compared to a life expectancy of 78.4 years, while in Czechia, the average age of the female COVID-19 deaths is 80.81, which is very close to the 82.1 life expectancy for women in that country).

These observations suggest that the impact of the pandemic in late 2020 on countries in Eastern Europe was not only large in absolute terms but also affected heavily younger demographics than in Western Europe.

One possible explanation for this discrepancy is the underlying comorbidity structure of population. Cardiovascular diseases (CVD) are a known risk factor for severe COVID, so we carried out a correlation analysis between the different excess mortality metrics we used and the prevalence of CVDs (based on 2018 data) in Europe. As a direct indicator for CVD prevalence we used CVDs death rates. We found strong correlation between PYLLs and CVD death rates, ASYRs and CVD death rates, and between WYLLs and CVD death rates restricted to age $<65$ years (Supplementary Fig. 9); correlation between EMR values/P-scores and CVDs was not statistically significant. These correlation warrant further investigation.

### Demographic-specific mortality patterns in Bulgaria

By the official statistics of the Ministry of Health^18,19^ the average age of a deceased male and female from COVID-19 are 69 and 71, respectively. The leading comorbidity is cardiovascular disease ($55\%$), followed by diabetes ($17\%$), pulmonary disease ($12\%$), obesity ($3\%$), and $30\%$ are listed with no known comorbidity. An overwhelming majority of $94.5\%$ of all 7,576 official COVID-19 deaths occurred in the hospitals with working age deaths comprising $28\%$ of all COVID-19 deaths. For the working age group females on average died at age 55.9 and males at age 55.7 with $45\%$ of the deceased having a cardiovascular disease.

Data on excess mortality for people under 65 reveals a slightly different picture. The working age group excess deaths are $21\%$ of all excess deaths with an average age of the deceased $55.65 \pm 0.07$ for men and $57.57 \pm 0.28$ for women. The reason for the higher average age for women is that our data does not reveal excess deaths in women under 40, whereas in the official statistics $5\%$ of the casualties are of ages between 10 and 39.

Next, we examine mortality in Bulgaria within the working age population in detail. Due to the well-documented age-related skew of COVID fatalities, we focused on two subgroups of working age individuals – those in the 30–39 and those in the 40–64 age ranges.

We find no elevated mortality in females in the 30–39 age group, while mortality is elevated in males of the same age bracket, with P-scores of –0.39 and 9.37, respectively (Fig. 4A–C).

In contrast, we find highly elevated excess mortality in both males and females in the 40–64 age group, in which Bulgaria and Lithuania rank highest in the EU (Fig. 4D–F), with P-scores of 23.2 and 28.6 for Bulgarian males and females, respectively. The difference between males and females is remarkable, as, unlike the typical situation, elsewhere in the world in this group in Bulgaria excess mortality measured by P-scores is lower for males than for females. We discuss the possible explanation for these observations in subsequent sections.

### Regional disparities in COVID pandemic-related mortality in Bulgaria

**Figure 5:**
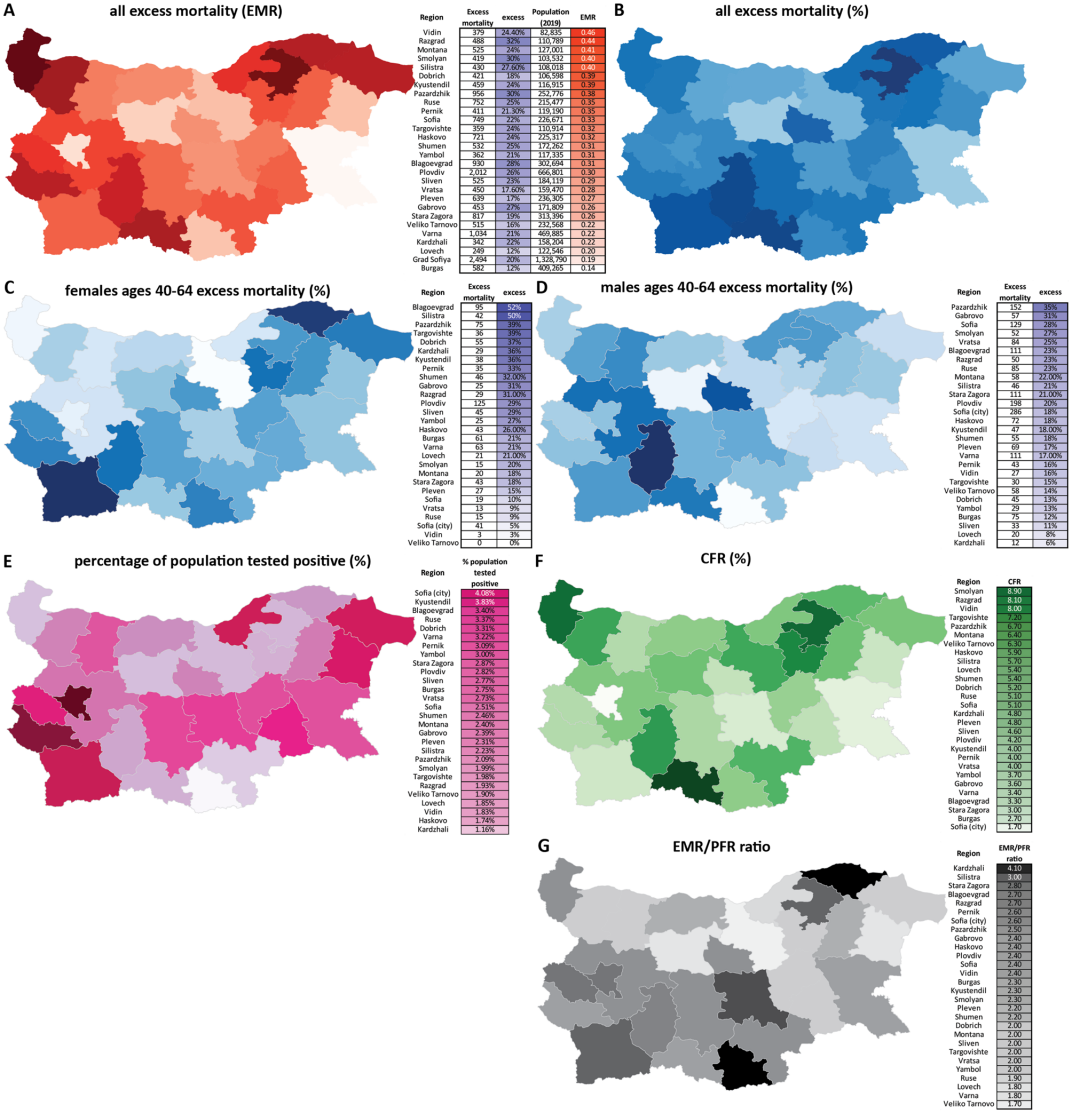
Regional disparities in the impacts of the COVID-19 pandemic in Bulgaria. (**A**) Overall excess mortality in Bulgarian regions (EMR units); (**B**) Overall excess mortality in Bulgarian regions (P-score); (**C**) Excess mortality in working age (40–64) females in Bulgarian regions (P-score); (**D**) Excess mortality in working age (40–64) males in Bulgarian regions (P-score); (**E**) Percentage of the population who have tested positive for SARS-CoV-2 in Bulgarian regions; (**F**) CFR values for Bulgarian regions; (**G**) Ratio between excess deaths (EMR) and official COVID-19-attributed deaths (PFR).

Following from the observation of considerable disparities between different European regions, we then analyzed regional differences in pandemic impacts within Bulgaria (Fig. 5). As a reminder, the overall statistics for Bulgaria are an EMR of $0.27\%$, P-score of $21.8\%$, CFR of $3.7\%$, an EMR/PFR ratio of 2.5, and a percentage of population tested positive of $2.9\%$.

The first major such disparity we observe is that between the four most populated provinces and the rest of the country. The excess deaths in these four major regions – Sofia (city), Plovdiv, Varna and Burgas – account for just $32\%$ of all excess deaths even though $\ge 50\%$ of the Bulgarian population lives there. Moverover, Sofia (city), Varna and Burgas have the lowest EMR of all provinces (Fig. 5A) and P-scores in the range 12–25% (Fig. 5B). The provinces of Sofia (city) and Burgas also show the two lowest CFR values (Fig. 5F).

In contrast, the more peripheral regions are among the most heavily affected. For example, the regions of Vidin and Silistra exhibit some of the highest EMRs – 0.46 and 0.40, respectively. Vidin also has the second highest CFR ($8\%$). The close to the average for the country P-score of $24\%$ in Vidin is likely a result of already very high pre-pandemic mortality in the region (the region has one of the fastest aging populations in the EU and the death rate there is 22 per 1000 people per year, whereas the death rate for Bulgaria is 15 deaths per 1000 people per year). In Silistra, the EMR/PFR ratio is 3.00, the second highest in the country and the P-score is $28\%$. In Kardzhali, the EMR/PFR ratio is 4.00, the highest in the country, and the P-score is $22\%$. With a P-score of $30\%$ and EMR of 0.40, Smolyan is one of the hardest hit regions in the country, and it also has a CFR of $8.9\%$, which is the highest among all provinces.

These regions also tend to show a lower percentage of the population that has tested positive, despite exhibiting the highest excess mortality (Fig. 5E), often high CFRs (Fig. 5F), and high EMR/PFR ratios (Fig. 5G).

We also find curious disparities in regional patterns of male- and female-specific excess mortality (Fig. 5C,D). The highest male excess mortality was observed in Pazardzhik, Gabrovo, Sofia (region) and Smolyan, while the highest female excess mortality is seen in Blagoevgrad, Silistra, Pazardzhik and Targovishte.

Remarkably, only $32\%$ of all excess deaths in the working age group occurred in Sofia (city), Plovdiv, Varna and Burgas. For women in the working age group only $29\%$ of the deaths occurred in those regions. A regional analysis reveals that the regions that with the highest P-scores in this demographic category are Blagoevgrad, Silistra, Pazardzhik, Targovishte, Kardzhali, Kyustendil and Dobrich ranging from $52\% \pm 6\%$ to $36\% \pm 7\%$ (Fig. 5C). Women in the age group 65–69, which includes working women in retirement age, were also heavily affected with an overall P-score of $23.7\%$ and exceptionally high regional P-scores in the provinces of Sliven - $76\% \pm 10\%$, Kardzhali - $46\% \pm 8\%$ Blagoevgrad - $45\% \pm 7\%$, Pazadzhik - $43\% \pm 6\%$, and Smolyan - $42\% \pm 11\%$ and (Supplementary Fig. 4). We discuss the possible explanations for these observations in the Discussion section.

**Figure 6:**
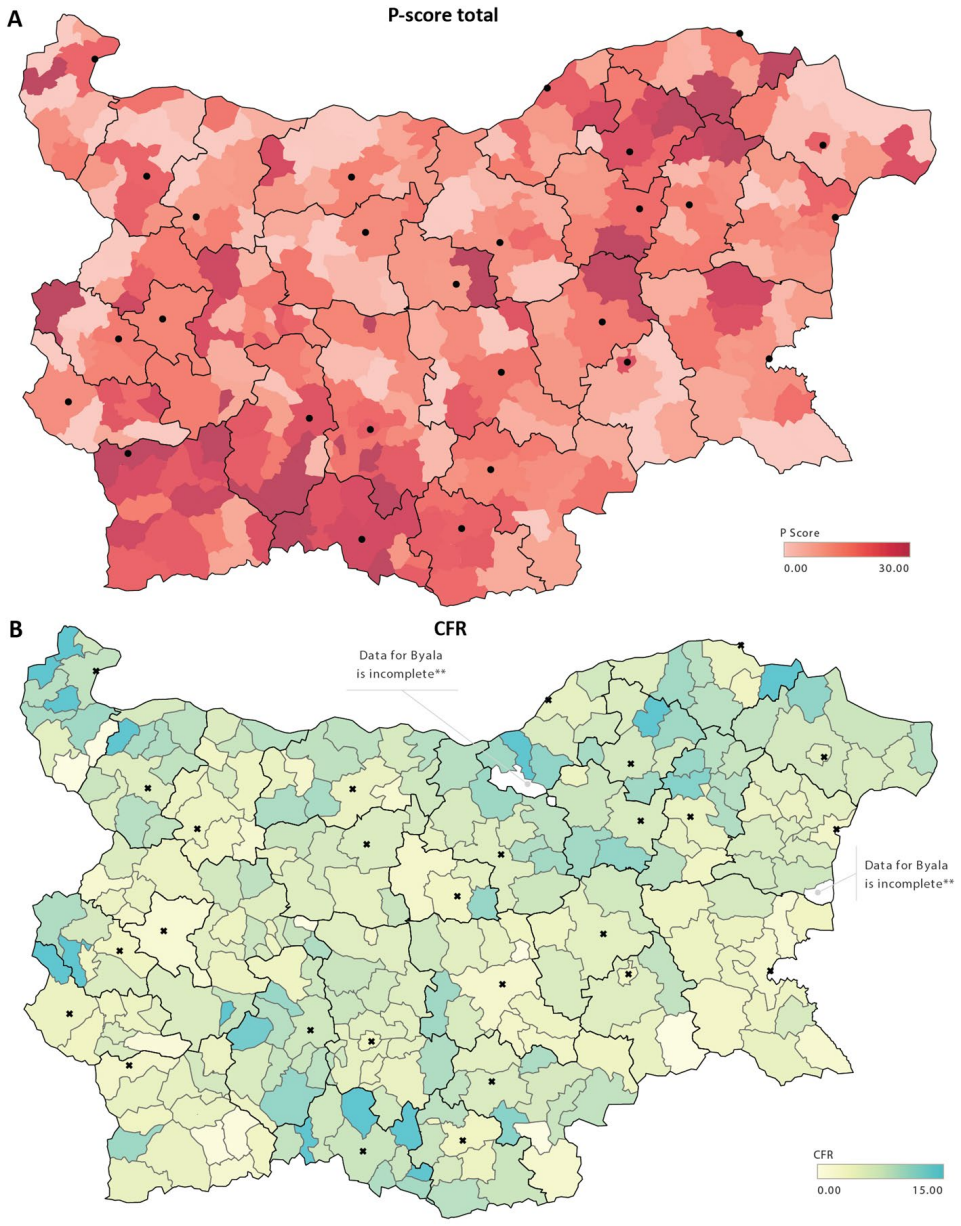
Regional disparities in the impacts of the COVID-19 pandemic in Bulgaria at the county/municipality level. (**A**) Overall excess mortality in Bulgaria at the county/municipality level; (**B**) CFR values for Bulgaria at the county/municipality level (note that there are two municipalities named “Byala”, and available data does not distinguish between the two, thus they are colored in white as missing data. Regions are demarcated in black, regional centers are shown as black dots.

These observations suggested that there might also be significant disparities in the impact of the COVID pandemic within regions, i.e. regional centers showing better outcomes than peripheral municipalities within each region. Examination of available data at the level of individual municipalities in Bulgaria indeed shows such as a pattern (Fig. 6, Supplementary Fig. 6).

CFR values are lower in regional centers, and the most heavily affected municipalities in each region tend to be smaller ones outside the region’s core city, and this is not an artifact of the their small population size. Some examples include: in Vidin, the region center’s P-score is 19.5% (±4.0%) while in Kula it is 39.4% (±10.5%); in Pernik, Pernik’s P-score is 16.3% (±1.3%) while in Tran it is 42.3% (±15.0%); in Gabrovo, Gabrovo’s P-score is 9.9% (±2.5%) while Tryavna has a P-score of 39.5% (±9.2%) and Dryanovo has a P-score of 36.3% (±9.4%).

**Figure 7:**
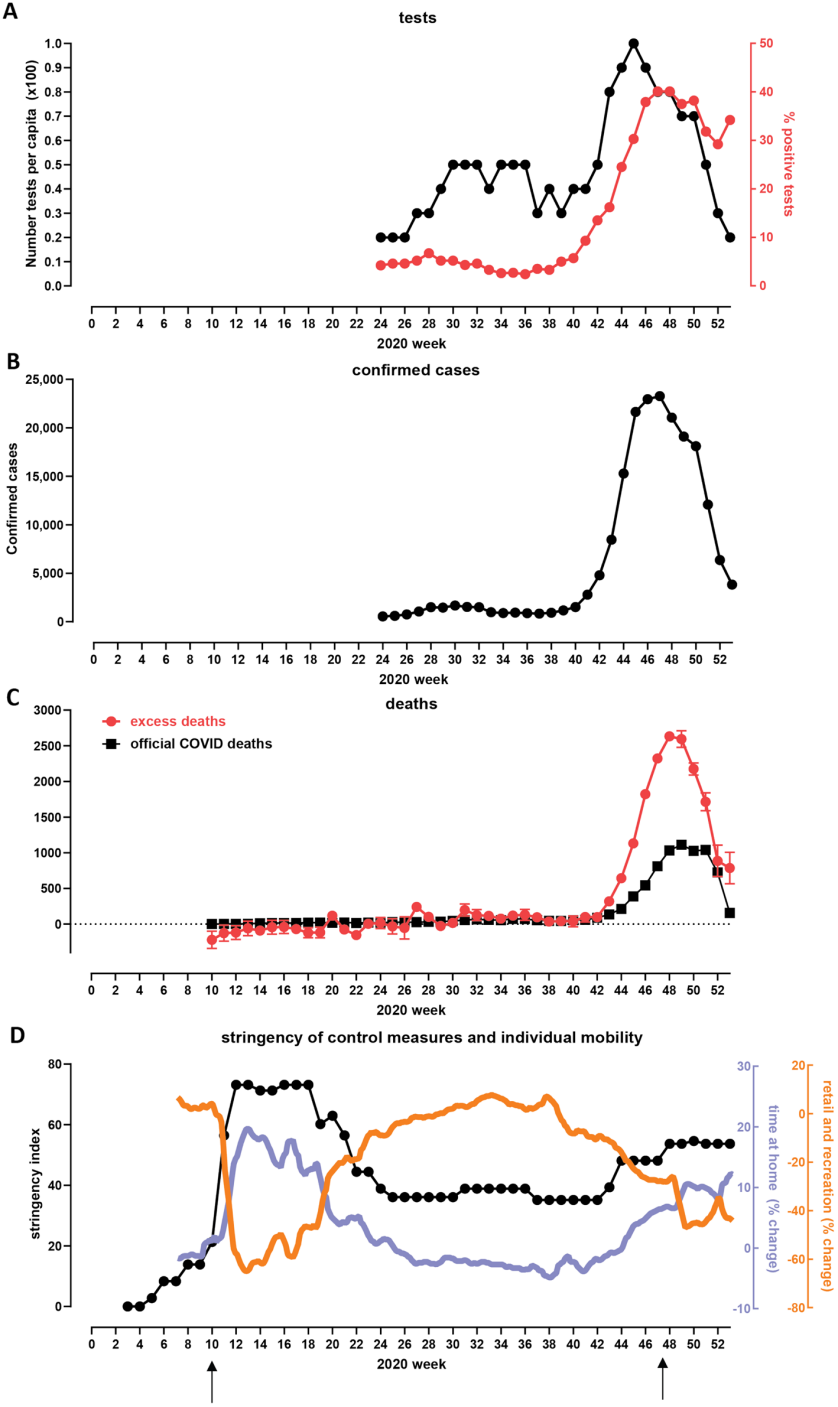
Development of the COVID pandemic in Bulgaria over 2020 and the effectives of measures implemented in order to control it. (**A**) Number of tests conduced and test positivity percentage over the second half of 2020. ^a^Official daily testing data was only made available from 06 Jun 2020–Open Data Portal (https://data.egov.bg/data/resourceView/e59f95dd-afde-43af-83c8-ea2916badd19). (**B**) Officially registered COVID cases. Note that rapid antigen tests were only included in statistics starting from December 22nd 2020. (**C**) Officially registered weekly COVID deaths and overall weekly excess mortality over the course of 2020. (**D**) Social mobility changes and the timing of imposition of restrictions. Arrows indicate the time of imposition of “lockdown” measures. “Time Home” refers to the change of the number of visitors to residential areas relative to the period before the pandemic. “Time Retail and Recreation” refers to the change of the number of visitors to places of retail and recreation relative to the period before the pandemic. This includes restaurants, cafes, shopping centers, theme parks, museums, movie theatres, libraries.

**Figure 8:**
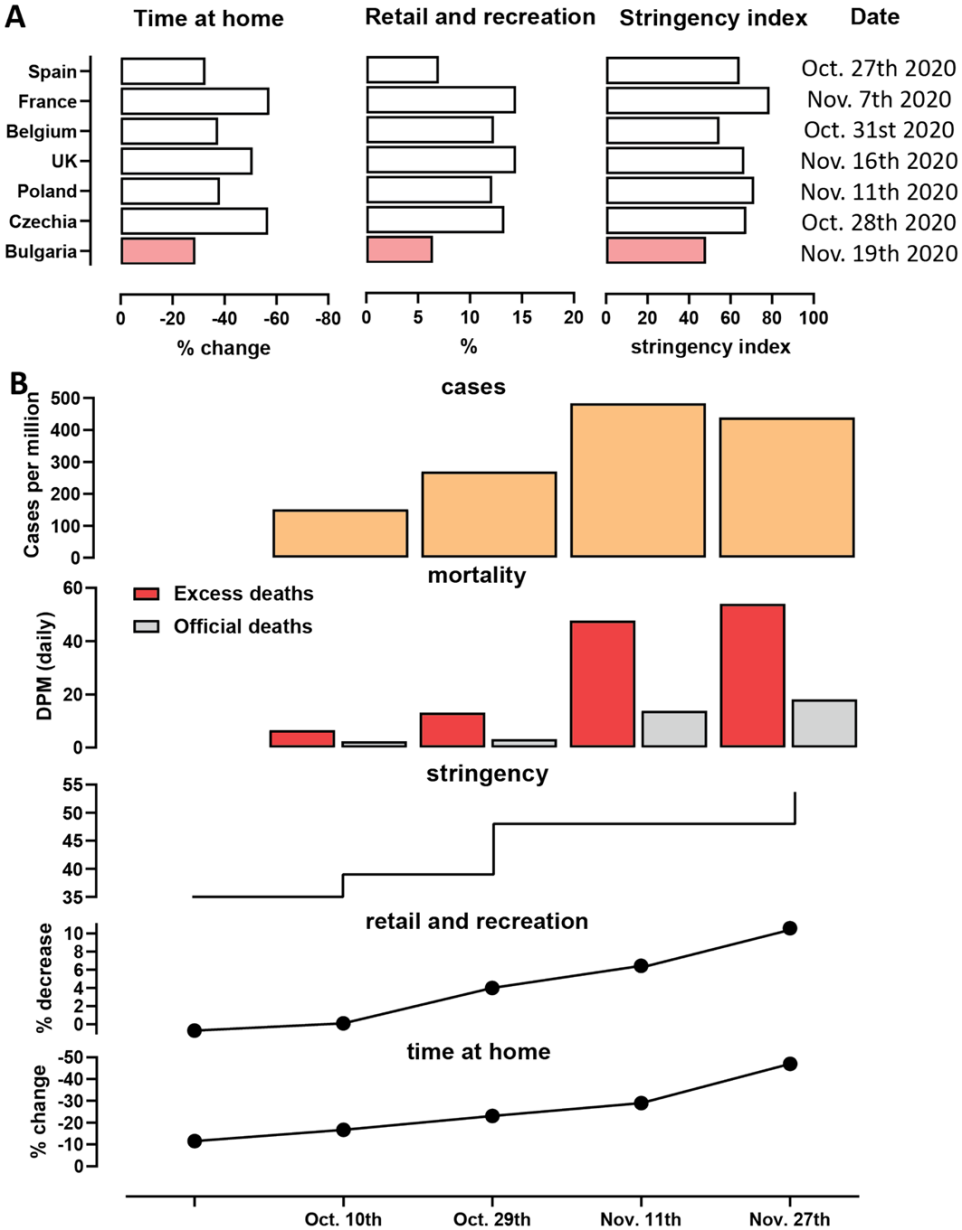
Mobility metrics, stringency of restrictions and mortality and cases at the peak of the late-2020 wave in Bulgaria. (**A**) Google Mobility Data and Stringency Index at the peak of the fall wave in Bulgaria and other EU countries. “Time Home” refers to the change of the number of visitors to residential areas relative to the period before the pandemic. “Time Retail and Recreation” refers to the change of the number of visitors to places of retail and recreation relative to the period before the pandemic. This includes restaurants, cafes, shopping centers, theme parks, museums, movie theatres, libraries. (**B**) Timeline of imposition of social distancing measures and of reductions in mobility in Bulgaria around the peak of the late-2020 wave. The peak occurred around November 11th 2020, as demonstrated by mortality data, which at any given moment reflects the dynamic of new cases in Bulgaria approximately 2.5 weeks prior to that moment.

### The trajectory of the pandemic in Bulgaria and the effectiveness of implemented pandemic control measures

Finally, we mapped the trajectory of the pandemic in Bulgaria onto the timeline of imposition of social distancing measures and independent measures of actual changes in societal mobility to understand the relationship between those factors and its development (Fig. 7).

The first period of the COVID-19 pandemic in Bulgaria, from March to the end of September, was marked by a slight elevation of the new confirmed cases in the summer, peaking at 242 cases per day towards the end of July. The excess mortality for this period is around 300 people and the official COVID-19 death toll amounts to 820 people, with a daily death rate of up to 10 deaths until the middle of October.

Rapid growth in the number of new confirmed cases started around the end of September. Then an explosion of cases occurred in late October, November and the first half of December (Fig. 7B). The peak in the 7-day moving average of the number of confirmed cases occurred on November 19th.

Official COVID-19 deaths peaked on December 6th with a 7-day moving average of 140, or ~18 DPM/day; excess deaths started decreasing around the same time (Fig. 7C). Excess deaths began diverging from official statistics with the start of the Fall surge, in the middle of October, and peaked at ~54 DPM/day in the week ending on November 27th. This corresponds to a $112.3\%$ increase in relative age-standardised mortality rates (rASMRs) according to the ONS^35^; a higher number in Europe in 2020 was observed only in Spain for the week ending on April 3rd at at $142.9\%$.

One of the obvious candidate explanations for the discrepancy between official and excess deaths is insufficient testing^36^.

Indeed, that appears to be the case for Bulgaria. Test positivity rates peaked ~40% in late November. However, a curious pattern is observed in the number of tests recorded in official statistics, which actually began decreasing while the positivity was still increasing in the month of November (Fig. 7A). An explanation for this pattern is that the results of rapid antigen tests were not included in official statistics until late December, and a considerable portion of testing shifted from PCR to antigen tests as the Fall wave developed. This likely accounts for at least some of the discrepancy between recorded and excess deaths.

We then examined the factors responsible for the Fall surge eventually receding using the stringency index and mobility metrics (see Methods), changes in which have been shown before to be predictive of the trajectory of COVID epidemics^37–40^.

The stringency index was at 35.19 from mid September until October 29th (Figs. 7D,8B), when the Bulgarian government imposed some new restrictions (high schools and universities moved to remote learning; nightclubs, pubs and bars were closed), which is reflected by an increase in the stringency index to 48.15. No further substantial epidemiological measures were introduced until after the peak of the fall wave – on November 27th, restaurants, bars, malls, schools and gyms were closed. However, the stringency index, though now increased to 53.7%, remained considerably below the levels of restrictions imposed in other European countries (Fig. 8A), as no stay-at-home orders or curfews were imposed, non-essential stores and hair-dressing salons remained open, and gatherings of up to 15 people were permitted.

As the peak of restrictions occurred around the time of the peak of excess mortality and thus after the peak of infections, it is likely that restrictions were not the main cause for the eventual decline in cases. Indeed, changes in people’s behavior as reflected in social mobility measures were observed much earlier than the imposition of restrictions, likely due to fear of becoming infected spreading among the population, a pattern previously noted elsewhere in the world^41^. Our data supports this: $40\%$ of the total increase from October 1st to November 27th of the time spent at home occurred in the period November 3rd to November 27th when no new substantial measures were introduced (see 8B).

The 7-day running average Google mobility data measured on November 19th shows a total decrease of at home compared to the baseline (Figs. 7D,8B). However, as with the stringency index, these values are still the lowest among analyzed European countries (Fig. 8B), which is likely a contributing factor to the very high excess mortality resulting from the pandemic.

**Figure 9:**
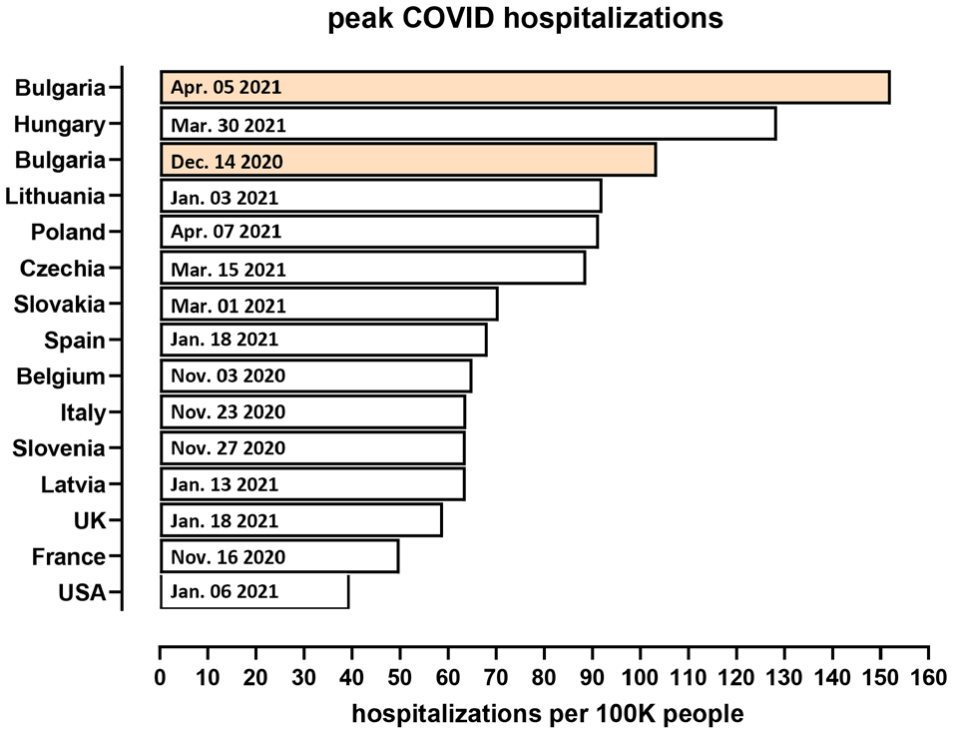
Peak hospitalizations in Bulgaria and other countries from the beginning of the pandemic till April 2021.

Finally, we examine hospitalization trends in Bulgaria and several other European countries. We find that at their peak on December 12th, hospitalizations in Bulgaria reached a level of $0.1\%$ of the population, which is one of the highest hospitalization rates up to date (it has since been exceed by Hungary and by Bulgaria itself during the subsequent March surge; Fig. 9).

## Discussion

In this survey, we analyze the impact of the COVID-19 pandemic in 2020 in Bulgaria and the broader Eastern European context. After a relatively low level of COVID-19 cases and deaths prior to that, in the concentrated span of less than three months in October, November, and December 2020, Bulgaria recorded the largest (per capita) number of excess deaths among the examined countries. Similar, though somewhat lower, large excess mortality increases were observed in most other countries in Eastern Europe. However, official COVID-19-attributed deaths account for only less than half of the excess deaths.

This discrepancy is likely caused by a combination of multiple factors – COVID-19 cases leading to death that were not reported as such in official statistics, COVID-19 cases resulting in death some time after recovery due to longer-term complications from the disease, and deaths from other causes that increased as a result to the inability of the healthcare system to treat them due to it being overwhelmed by COVID-19 patients. As the disparity between excess deaths and official COVID-19 mortality is very large in the case of Bulgaria – excess deaths amount to ~0.27% of the population while official COVID-19 are at ~0.11%, i.e. a ~0.16% difference – and excess mortality is highly temporally concentrated in a short time span of about ten weeks (i.e. the contribution of the latter two factors is unlikely to have been so large in such a brief period), it is most likely that the bulk of excess deaths were caused directly by COVID-19.

Why they were not recorded as such is also probably due to a multitude of factors. Testing in Bulgaria has been greatly insufficient throughout the pandemic and even more so during the late-2020 surge and the lowest among the examined countries (Supplementary Fig. 8; in addition to that, the decision to not include rapid antigen tests in public statistics certainly has contributed to the underreporting. Most reported deaths occurred in hospitals, and many of those who could not be hospitalized due to healthcare systems being overwhelmed and died at home were not recorded as having died of COVID. Whether additional social and socioeconomic factors could have contributed, as has been suggested to be the case elsewhere in the world^42^, is a subject for future investigations, as is the question of whether the reasons for underreporting are uniform across the more general Eastern European region. Lack of testing on its own in turn has probably contributed to the epidemic growing out of control and leading to such a number of excess deaths.

Another contributing factor to the high mortality rate in Eastern Europe is probably the very high prevalence of cardiovascular diseases in the region^43^ also suggested by our correlation analysis in Supplementary Fig. 9. In Bulgaria over half of the COVID-19 officially reported fatalities are listed with cardiovascular disease as a comorbidity.

Bulgaria also exhibits one of the most highly elevated working-age excess mortality, and it is also an outlier in terms of working-age excess mortality among females. We also observe significant regional disparities within the different regions in the country in total and in working age sex-specific excess mortality. A possible explanation for the latter is the development of outbreaks at workplaces where mostly women work – for example, garment, textile and shoe factories, which in Bulgaria almost exclusively employee women and which are major sectors of the economy in provinces such as Blagoevgrad, Kardzhali, Smolyan, Sliven, and Kyustendil^44^. Indeed, there were numerous reports about outbreaks in such settings. Analogous causality might be behind regional disparities in working age male-specific excess mortality (Fig. 5D). A list of reports about outbreaks in these regions can be found in our GitHub repository, which includes reports about outbreaks in battery, automotive parts, power transmission, sanitary ceramics, and other factories.

Regional disparities in overall excess mortality, in particular the clear dichotomy emerging between the major population centers, in which generally better outcomes are observed, and the more heavily affected peripheral regions, also warrant further investigation. COVID-19 is still often considered a disease that impacts highly populated big cities the most, where disease spread is thought to be facilitated by density; this is due to many of the most notable initial outbreaks affecting well-connected in terms of international travel large metropolitan areas. However, as the pandemic has spread throughout the countries that have not controlled it, it may be the case that previously established regional disparities in healthcare infrastructure are becoming a key factor determining differential outcomes between generally better resourced major cities on one hand, and the less equipped to test, track and treat COVID-19 patients countryside areas. There is evidence that such causation is at play in Bulgaria – many of the heavily affected regions have fewer ICU beds, fewer doctors, and fewer specialists in the most relevant to the treatment of COVID-19 specialties than the capital and a few other major cities (Supplementary Fig. 7). For example, Vidin and Silistra have fewer than average hospital beds, Kardzhali has the lowest number of doctors, general practitioners and pulmonologists and the second to last number of ICU beds per capita in the country, and Smolyan has the lowest number of ICU beds (just 9 in total for the whole region) and a generally low number of doctors.

In addition, in some of these regions (e.g. Smolyan, one of the most heavily impacted in the country) there are purely geographic factors that may have complicated the timely treatment of patients due to the logistic challenges of transporting patients to the regional center (which is where the only ICU units are located) from remote small towns through mountainous terrain while the core city’s health infrastructure is itself under immense stress (as shown in Fig. 9, Bulgaria recorded record hospitalization levels during the peaks of the pandemic). For example, four of the peripheral municipalities in the Smolyan region have twice as high CFR values as the city of Smolyan (see Fig. 6B). Whether similar regional patterns of pandemic-related excess mortality are observed in other areas of Europe will be informative and instructive for minimizing the impact of subsequent COVID-19 waves.

It should also be noted that healthcare disparities possibly play a role on a broader-level^45–47^, as Eastern Europe’s healthcare systems as a whole are well-documented to be suffering from an outflow of skilled medical labor due to large numbers of doctors and nurses emigrating to Western Europe in recent years^48^.

However, the main factor behind the very high levels of excess mortality is still most likely the late imposition of restrictions on social mobility and lax governmental efforts at controlling the spread of SARS-CoV-2, as our analysis shows. In Bulgaria these were adopted long after exponential growth in cases had commenced and was clearly going to overwhelm hospital resources, little testing was carried out and insufficient efforts were made to ensure the isolation of infected individuals, and even when restrictions were imposed, they were generally the most lax in Europe; furthermore, the late-2020 epidemic appears to have begun to trend downward due to changes in individual behavior, the onset of which actually preceded the imposition of restrictions by the government. The high levels of excess mortality are probably a natural consequence of following these policies.

## Methods

### Data sources

All-cause mortality data for European countries, as well as NUTS-3 (Nomenclature of Territorial Units for Statistics) regions in Bulgaria, was obtained from Eurostat^11,12^. The data presented in the datasets is sex- and age-stratified, with age groups split in increments of 5 years. Since not all countries submit data at the same time and in the same manner, only countries that have consistent weekly data for the period 2015–2020 (inclusive) were analyzed.

Country-level population data at the beginning of 2020 was collected through Eurostat^13^, but was further supplemented by population data from the United Nations’ UNdata Data Service^14^. We further elaborate on this topic in the subsequent section on Potential Years of Life Lost (PYLL) and Working Years of Life Lost (WYLL) estimates.

Life expectancy values at different ages were obtained from three separate sources. We acquire the full life tables for Bulgaria through the country’s National Statistical Institute^15^, and for Czechia through the country’s Statistical Office^16^. Abridged life tables for all European countries were obtained from the World Health Organization’s open data platform^17^. This dataset is partitioned by age, in increments of 5 years.

COVID-related mortality and testing data for Bulgaria was collected through the resources available from the Ministry of Health^18,19^. COVID-related mortality for Czechia was acquired from Czech Ministry of Health official website tracking the pandemic^20^.

### Excess mortality and P-scores

To calculate excess mortality across countries as well as across Bulgarian regions, we analyze the mortality observed between week 10 and 53 of 2020 and compare it to expected (baseline) mortality for 2020 using the historical data for the previous five years (2015–2019). The model we used is the Karlinsky–Kobak regression model^6^:

$$\begin{aligned} D_{t,Y}=\alpha _t +\beta \cdot Y + \epsilon \end{aligned}$$

where $D_{t,Y}$is the number of deaths observed in week or month* t* in year* Y*, $\beta$ is a linear slope across years, and $\alpha _t$ are separate intercepts (fixed effects) for each week or month and $\epsilon \sim {\mathcal {N}}(0,\sigma ^2)$ is Gaussian noise. The model prediction for 2020 is ${\text {Expected\;Mortality}}_{t,2020}=\hat{\alpha _t}+\hat{\beta }\cdot 2020$. We then establish a 95% confidence interval for the expected mortality. This range is used to calculate the excess mortality $\Delta _t$ for a week or a month* t* as:

$$\begin{aligned} \Delta _t= \text {Mortality}_{t,2020} -\text {Expected Mortality}_{t,2020}. \end{aligned}$$

This calculation is done both as a sex- and age-stratified metric, as well as an aggregated total excess mortality for 2020, which we denote by $\Delta$. To normalize excess mortality across countries, we calculate excess mortality per total population. To do this we use population data from Eurostat for 2020. 

Set $z:= |\Delta |/\sqrt{\mathrm{Var}[\Delta ]},\, \text {where}\, \hbox {Var}[\Delta ]$is computed in^6^. If $z$ is significantly below 2 for a given country, we consider the excess mortality for this country to be not significantly different from zero. In the computations related to the years-of-life lost metrics considered in the paper, we excluded a few countries having both $z$-values significantly below 2 (typically lessthan 1) for each age interval and wide confidence intervals that included 0 for the excess mortality associated with each of these age intervals.

Based on the excess mortality ranges we also compute a P-score value for each country/region. A P-score value is defined as the ratio or percentage of excess deaths over certain period relative to the expected deaths for the same period based on historical data from the years 2015–2019 (see^21^). We calculate the P-score as follows:

$$\begin{aligned} P:=\frac{\hbox {Mortality}_{2020} - \text {Expected Mortality}_{2020}}{\text {Expected Mortality}_{2020}} * 100 \end{aligned}$$

We also calculate the ratio between excess mortality and official COVID-19-attributed mortality. Due to the demonstrably low testing in Bulgaria^22^ and other countries, this allows us to estimate under-reported COVID-19 fatalities. We also use the total positive tests per region reported at the end of 2020 to compute a Case Fatality Ratio (CFR) which estimates the proportion of COVID-19 fatalities among confirmed cases.

### Potential Years of Life Lost (PYLL), Aged-Standardized Years of life lost Rate (ASYR), and Working Years of Life Lost (WYLL) estimates

Potential Years of Life Lost (PYLL) is a metric that estimates the burden of disease on a given population by looking at premature mortality. It is derived as the difference between a person’s age at the time died and the expected years of life for people at that age in a given country. As such, the metric attributes more weight to people that have died at a younger age.

We compute the PYLL across countries by taking the positive all-cause excess mortality for all ages groups (in Eurostat they are aggregated at 5 year intervals). We use the abridged life expectancy tables by the WHO (also aggregated at 5 year intervals) and calculate a total and average PYLL value for all countries. To be more precise, for an age interval $[x,x+4]$ and sex* s* (if no sex is specified we assume it’s for both sexes) define by $\hbox {ED}([x,x+4],s)$ the excess deaths and by $\hbox {LE}([x,x+4],s)$ the life expectancy. Then the potential years of life lost are computed as

$$\begin{aligned} \hbox {PYLL}([x,x+4],s)=\hbox {ED}([x,x+4],s)*\hbox {LE}([x,x+4],s). \end{aligned}$$

The total PYLL is computed by summing over all age intervals. In our computations we take into account the margin of error for each $\hbox {ED}([x,x+4],s)$.

A limitation on this approach is the upper-boundary aggregation value for the two datasets. The all-cause mortality dataset’s upper boundary is 90+, while the WHO’s abridged life tables only go up to the 85+ age bracket. To account for this, we attribute the life expectancy of the 85+ age group to the 85-89 mortality group. We have further excluded the 90+ mortality group from our analysis. This is further elaborated on in the Limitations subsection, where we also provide a way of correcting for this exclusion.

Two countries for which we have the exact ages and sex for each reported COVID-19 fatality are Bulgaria and Czechia. We also have full life tables (increments of one year) for both countries provided by their respective statistical institutes. This allows us to compute and compare the PYLLs for each country based on excess mortality data and official data for COVID-19 fatalities.

Finally, we standardize PYLL values across countries by diving the total sum value by the population and normalizing it per 100,000 people:

$$\begin{aligned} \hbox {PYLL}_{\mathrm{std}}:=\frac{\hbox {PYLL}_{\mathrm{total}}}{\text {Total Country Population}_{0-89}} * 100,000 \end{aligned}$$

The data for country-level populations in Eurostat has a similar limitation in the upper boundary of the age distribution (a cut-off at 85+). To mitigate this limitation, we supplement the population data from Eurostat for ages 0-84 with population size data for the 85-89 age group from the UNdata Data Service.

To compare the impact of the pandemic across European populations with different age structures we compute the Age-Standardized Years of Life Lost Rate (ASYR)^23,24^. Let $([x,x+4],s)$ as be an age interval for a sex* s* in a standard life expectancy table for a given population. Denote by $P([x,x+4],s)$ the population size of $P([x,x+4], s)$. Define the PYLL rate for $([x,x+4],s)$

$$\begin{aligned} \hbox {PYLL}_{\mathrm{rate}}([x,x+4],s) :=\frac{\hbox {PYLL}([x,x+4])}{P([x,x+4],s)}*100,000. \end{aligned}$$

For the 2013 European Standard Population (ESP) denote by $W([x,x+4],s)$ the weight of $([x,x+4],s)$ in the standard population. Define

$$\begin{aligned} \hbox {ASYR}(s):=\sum \hbox {PYLL}_{\mathrm{rate}}([x,x+4],s) * W([x,x+4],s) \end{aligned}$$

where the sum is taken over all age intervals. For a given population of sex *s* this measure is interpreted as the years of life lost per 100,000 people (of sex *s*) if the population has the same aged is tribution as the ESP. ASYR allows for comparison of the pandemic impact on EU countries having different age distributions. Finally, we derive total average and total standardized WYLL value approximations. To accomplish this we first assume people to be in the working age group if they are 15 to 64 years old, and thus exclude excess mortality for all age groups over 65. To calculate the remaining years of working life, we further assume a mean age for each age group, e.g. for the age interval 60–64 we assume a mean age at 62.5 years. This would leave this group with approximately 2.5 years until retirement. Limitations on this approach are discussed in the subsequent Limitations subsection.

### Stringency index and mobility data

Metrics of population mobility were obtained from the Google COVID-19 Community Mobility Reports^25^. These datasets contain data on how visits and length of stay at different places change compared to a baseline by generating anonymized metrics from data of Google users who have switched on “Location History” on their mobile devices.

To quantify governmental pandemic-response measures across countries, we used the Oxford COVID-19 Government Response Tracker^26^, which systematically collects information on several different common policy responses that governments have taken to mitigate the effects of the pandemic^27^. This allows a comparison of governmental measures between over 180 countries worldwide.

### Limitations

Each of the presented data sources and approaches to analysis have their own limitations. Below we discuss each one in detail.

#### Limitation of scope

The current time frame that is analyzed creates a hard boundary between week 10 and week 53 of 2020. The exit conditions of different countries at these boundaries, however, are not equal. Some countries experienced subsequent surges in January 2021 and later months. Thus the current research provides a snapshot of the effects of the pandemic up to the end of 2020, not the totality of its effects.

#### Limitation of data

All cause mortality figures for 2020 are still provisional for most EU countries, so they are subject to readjustment in future time. Even so, they can provide a good estimate of the effect of COVID-19 in different countries up to this point.

#### Limitation of excess mortality and P-scores

Influenza outbreaks in the period 2015–2019 contribute to the estimation for the expected mortality for 2020. Thus the expected mortality is an estimate of the “normal” death rate in the presence of seasonal influenza.

Since the P-score metric we compute is derived from the excess mortality figures we calculate for each individual country, this metric also suffers from the issues we outline for excess mortality.

#### Limitations of PYLL/ASYR/WYLL

Since PYLL, ASYR and WYLL data only take into account fatalities, these metrics do not provide information about any worsened quality of life of surviving individuals, reduced life expectancy of these individuals and working capacity. Metrics such as Disability-Adjusted Life Years (DALY), Quality-adjusted life year (QALY) and Healthy Years of Life (HALE) metrics may illuminate further the total disease burden on the European population, however, obtaining the necessary information for these measurements is not yet possible.

As mentioned before, due to data availability limitations from Eurostat in our computations of PYLLs and ASYRs we excluded the 90+ group. Given that countries like France, Italy and Spain have significant excess mortality in this age group we also present a computation of the ASYRs including the 90+ age by assuming 4 years of life expectancy (the average life expectancy for the 90+ age group for the European populationis 4.74, according to the UNdata Data Service). By linear interpolation, 4 is approximately the life expectancy for the age interval [90,94] assuming that the average age of deaths for this interval is in the range 92.7-93 (the average age of the COVID-19 fatalities above 90 years of age is 92.7 in Czechia and 92.1 in Bulgaria, and likely it’s higher in Western countries which overall have higher life expectancy).

This rough approximation gives an upper bound of how large the ASYRs can go. It leads to 5–14% and 14–22% increase in the ASYRs for the $(0-89)$ population of Eastern and Western European countries, respectively, but it does not yield a decrease between the inequalities of the countries from the two groups or any significant change in their ranks (see Supplementary Fig. 3).

The WYLL measure we present has some additional limitations. The first comes from the assumption that retirement age across European countries is 65. While it is most often assumed as a standard between European countries, there is actually some variation between individual member states^30^. Furthermore, we assume that the mean age of people who have died in a given age group is the middle of the given range, e.g. for the age group 60–64 - $\text {mean age} = 62.5$. It may well be a fact that a majority of the fatalities are concentrated in the upper part of the age bracket. However, since we do not have data about the different causes of mortality, but rather an aggregate total, we cannot be certain that this trend will hold true for all age groups and across different countries.

#### Google COVID-19 community mobility reports

Bulgaria is below the EU average when it comes to use of mobile devices in the 16–74 age group. Still, a majority of the population within that group (~64%) utilized mobile devices to access the internet in 2019^31^. However, it is possible that there might be a skew towards the younger half of this age range of users who are supplying data.

## Supplementary Information

Supplementary Information.

## Data Availability

All datasets and associated code can be found at https://github.com/Mlad-en/COV-BG.git.
