# Publisher Correction: The demographic and geographic impact of the COVID pandemic in Bulgaria and Eastern Europe in 2020

**DOI:** 10.1038/s41598-022-11420-4

**Published:** 2022-04-29

**Authors:** Antoni Rangachev, Georgi K. Marinov, Mladen Mladenov

**Affiliations:** 1 Department of Mathematics, University of Chicago, Chicago, IL 60637 USA; 2 Institute of Mathematics and Informatics, Bulgarian Academy of Sciences, Sofia, 1113 Bulgaria; 3 Department of Genetics, Stanford University, Stanford, CA 94305 USA; 4 Premier Research, Morrisville, NC 27560 USA

Correction to: *Scientific Reports* 10.1038/s41598-022-09790-w, published online 15 April 2022

The Supplementary Information file published with this Article was incorrect as it was the manuscript file. The correct Supplementary Information file now accompanies the original Article.

