# Supplementary material for: The demographic and geographic impact of the COVID pandemic in Bulgaria and Eastern Europe in 2020
Source: Sci Rep. 2022 Apr 15;12:6333. doi: 10.1038/s41598-022-09790-w (PMC9012259; doi:10.1038/s41598-022-09790-w)
Supplement: Supplementary file 1 — Supplementary Information. [file 41598_2022_9790_MOESM1_ESM.pdf]

Supplementary Materials

Supplementary Figures

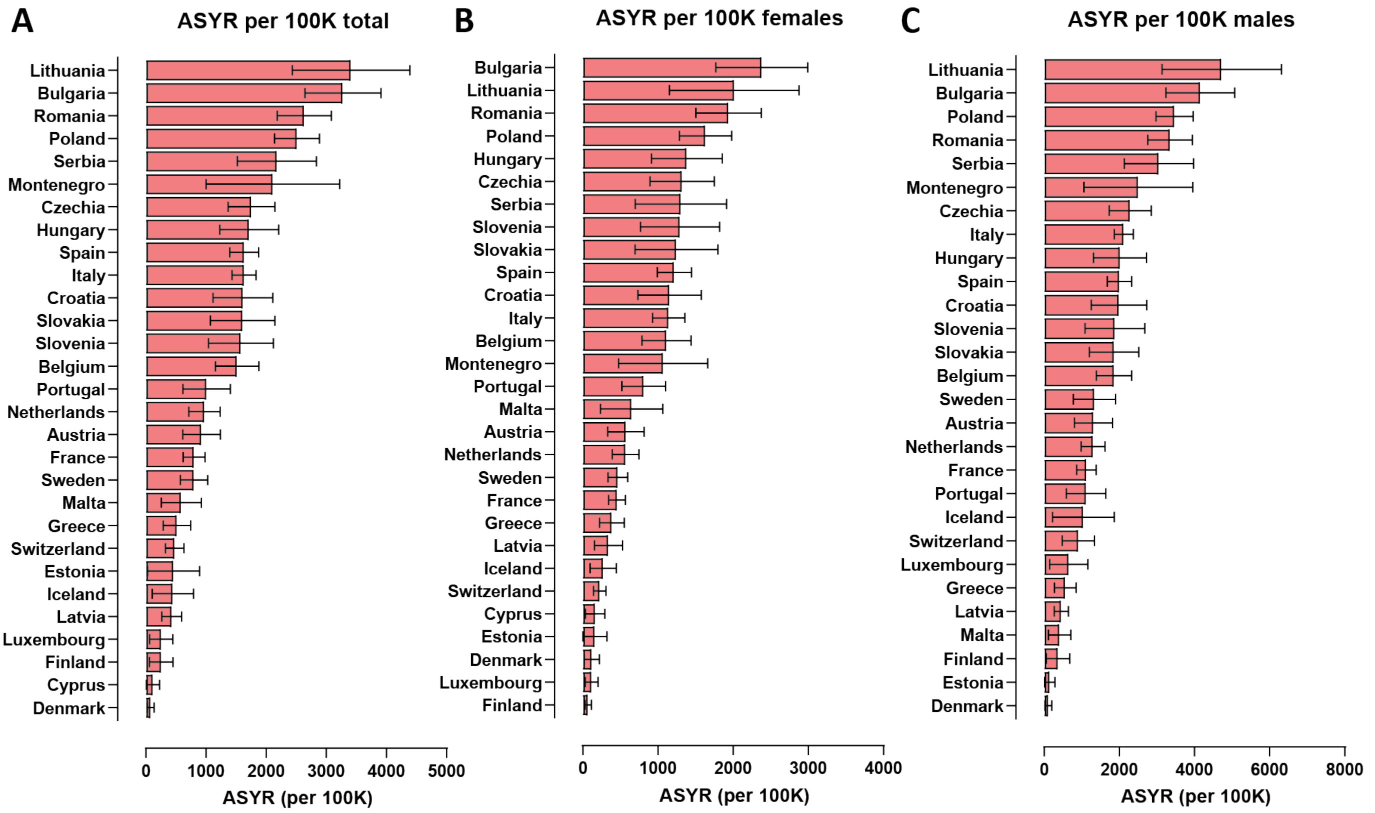

Supplementary Figure 1: Excess mortality-based ASYR values for European countries in 2020. Shown are the total (per 100K people) values. (A) ASYR values for the whole population; (B) ASYR values for females; (C) ASYR values for males.

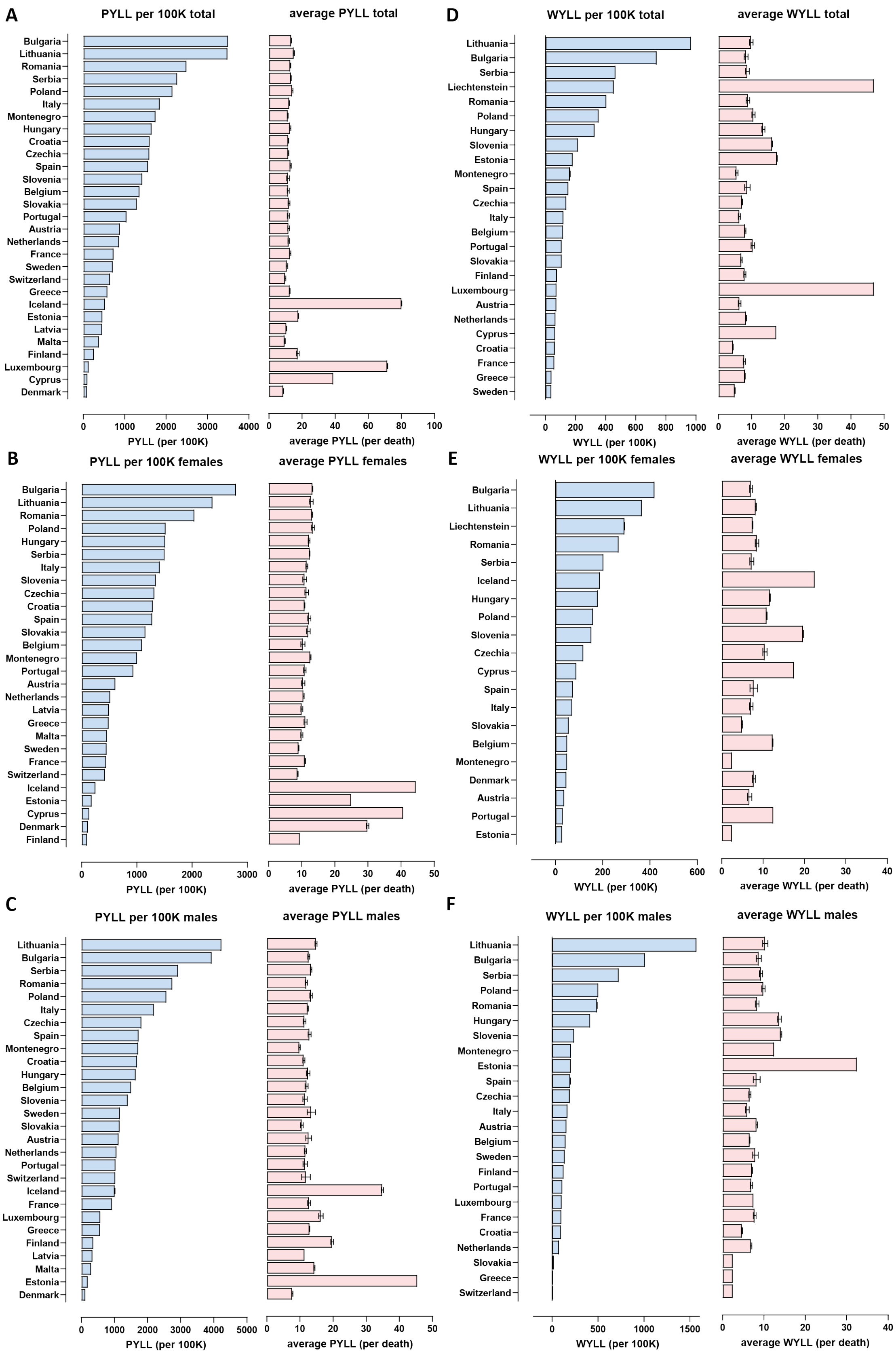

Supplementary Figure 2: Excess mortality-based PYLLs and WYLL values for EU countries in 2020. Shown are the total (per 100K people) and average (per death) values. (A) PYLL values for the whole population; (B) PYLL values for females; (C) PYLL values for males; (D) WYLL values for the whole population; (E) WYLL values for females; (F) WYLL values for males.

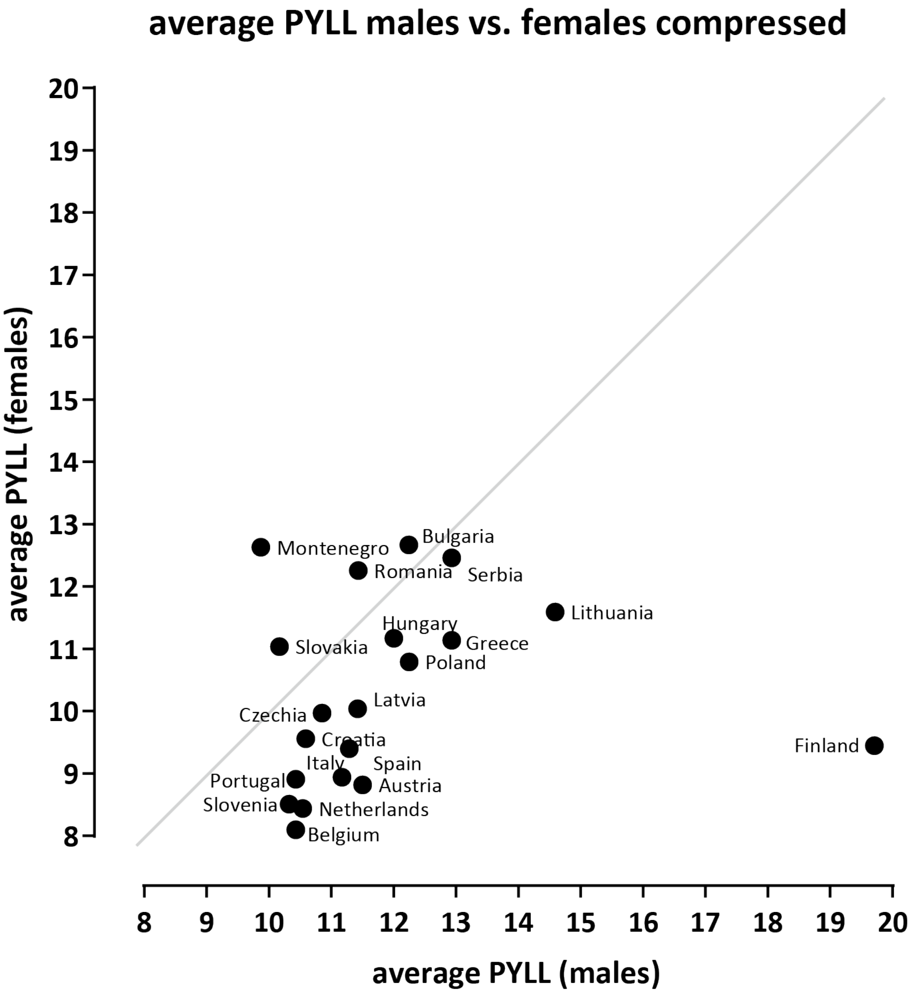

Supplementary Figure 3: Comparison between male- and female-specific average PYLL values in European countries. Shown are the average PYLL values per excess death.

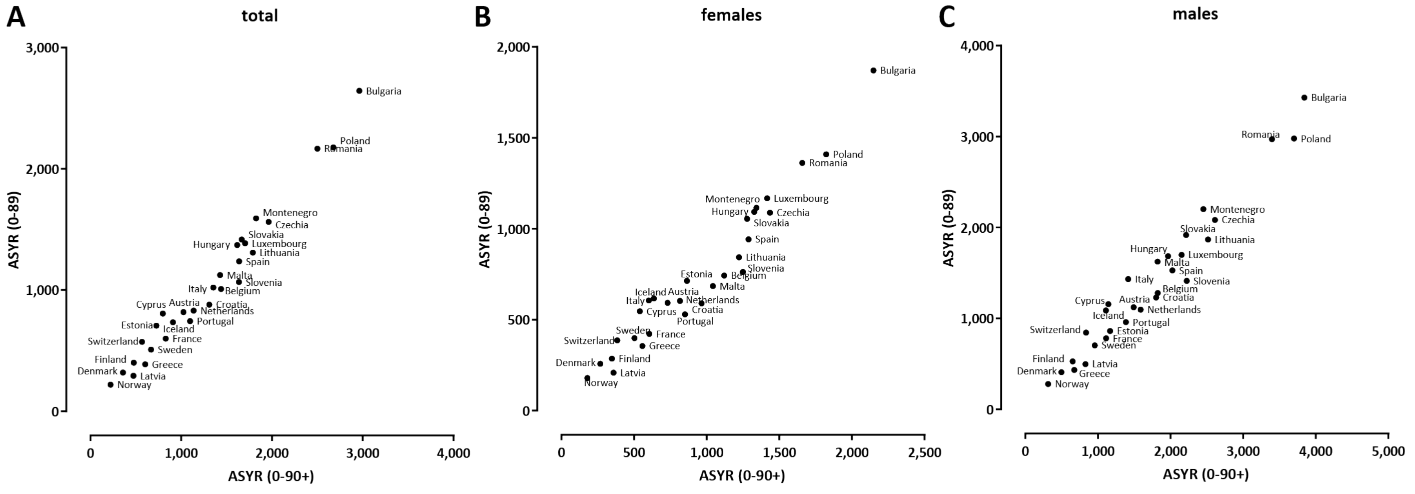

Supplementary Figure 4: ASYR values computed over the 0-89 age range and ASYR values computed over the whole population (with 90+ year-olds included) are consistent. (A) ASYR total; (B) ASYR females; (C) ASYR males.

A

females ages 65-69 excess mortality (%)

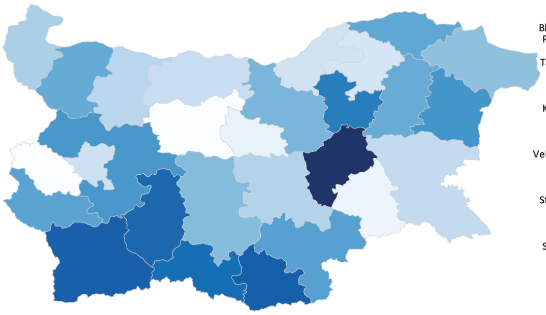

Region	Excess mortality	excess
Sliven	62	76%
Kardzhali	24	46%
Blagoevgrad	49	45%
Pazardzhik	50	43%
Smolyan	19	42%
Targovishte	23	39%
Varna	61	34%
Sofia	41	33%
Haskovo	33	31%
Kyustendil	19	30%
Silistra	18	29%
Montana	22	29%
Shumen	25	28%
Veliko Tarnovo	32	26%
Plovdiv	64	24%
Dobrich	23	23%
Vidin	12	19%
Stara Zagora	26	17%
Vratsa	15	16%
Burgas	24	14%
Pleven	17	13%
Sofia (city)	61	12%
Ruse	12	11%
Razgrad	6	10%
Gabrovo	3	5%
Yambol	2	4%
Pernik	-1	-2%
Lovech	-10	-13%

B

males ages 65-69 excess mortality (%)

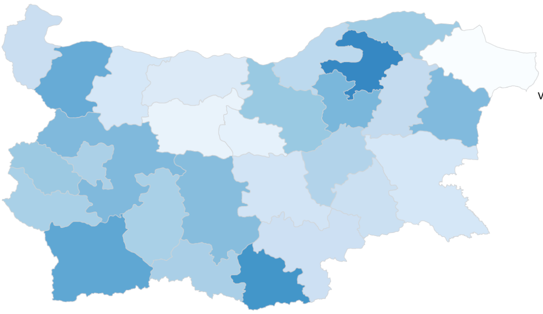

Region	Excess mortality	excess
Razgrad	44	37%
Kardzhali	34	34%
Blagoevgrad	65	29%
Montana	44	28%
Targovishte	27	26%
Sofia	60	25%
Varna	84	25%
Plovdiv	121	24%
Veliko Tarnovo	46	21%
Pernik	26	21%
Silistra	23	20%
Pazardzhik	43	19%
Kyustendil	27	19%
Smolyan	18	19%
Sofia (city)	148	18%
Sliven	30	17%
Ruse	32	16%
Shumen	22	14%
Vidin	13	13%
Yambol	16	13%
Haskovo	26	12%
Stara Zagora	29	11%
Vratsa	18	10%
Burgas	31	10%
Pleven	19	8%
Gabrovo	6	5%
Lovech	6	5%
Dobrich	-10	-5%

Supplementary Figure 5: Excess mortality in the 65-69 age range in Bulgarian regions. (A) females, P-scores; (B) males, P-scores.

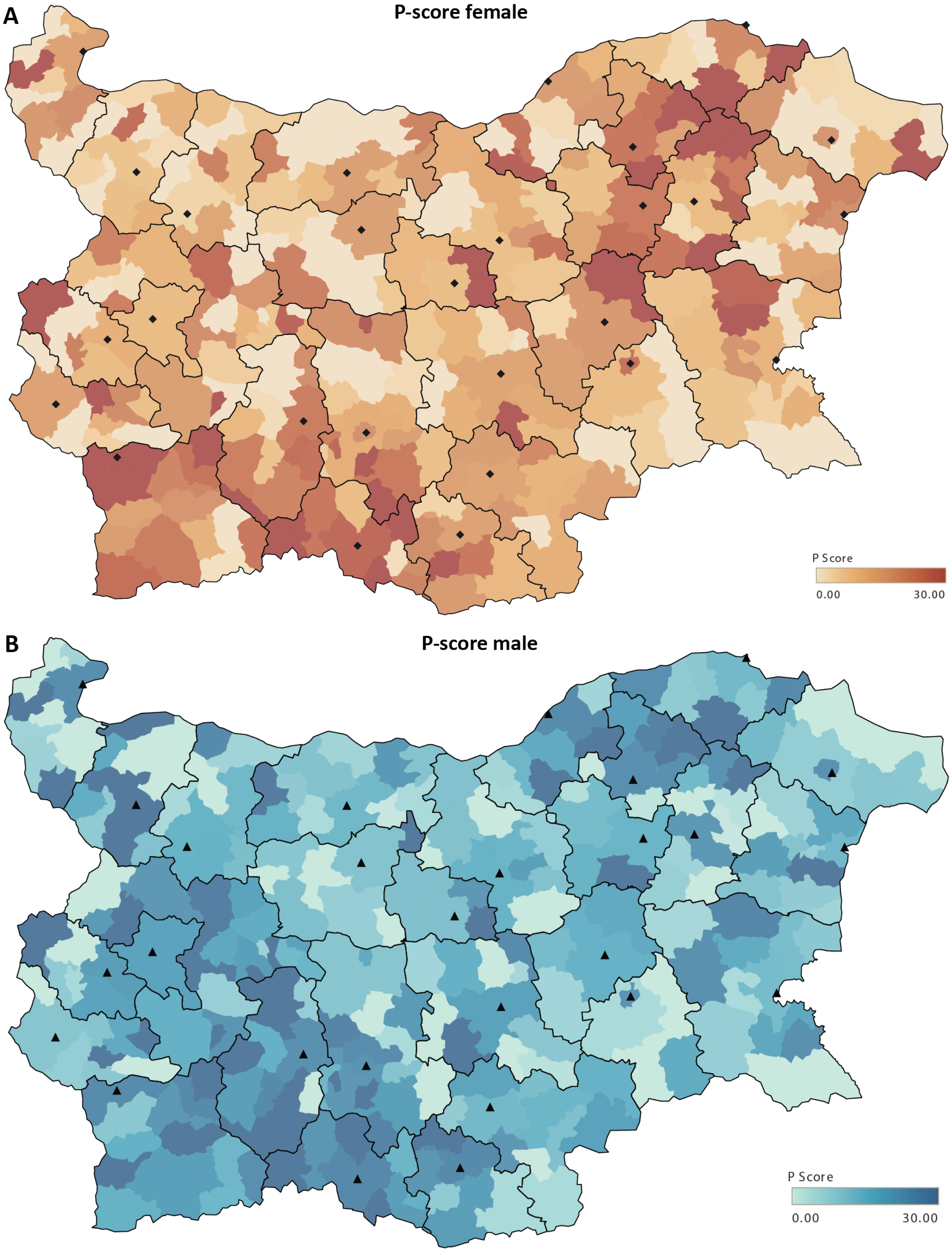

Supplementary Figure 6: Regional disparities in the impacts of the COVID-19 pandemic in Bulgaria at the county/municipality level. (A) Female excess mortality in Bulgaria at the county/municipality level; (B) Male excess mortality in Bulgaria at the county/municipality level; Regions are demarcated in black, regional centers are shown as black dots.

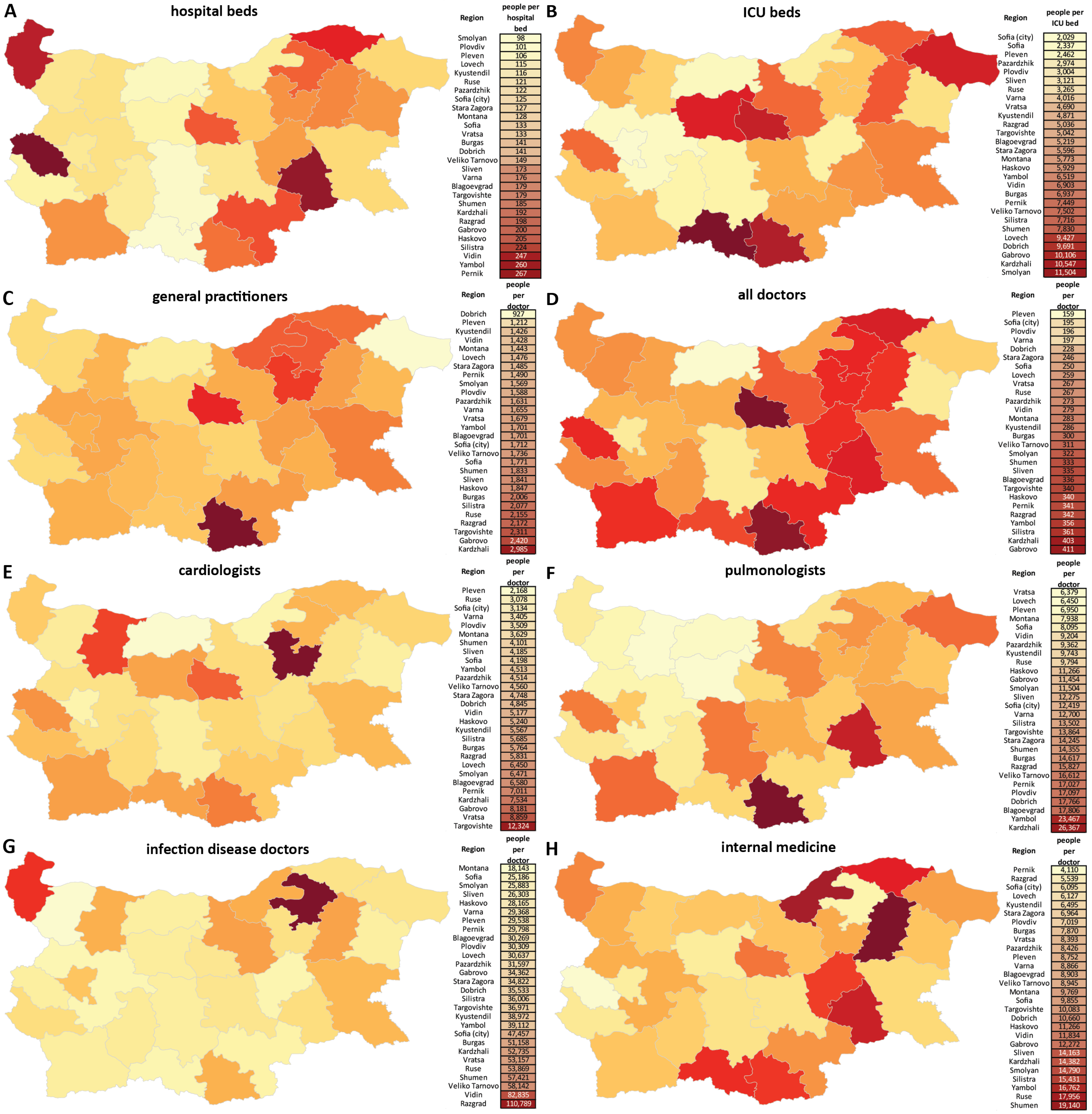

Supplementary Figure 7: Availability of medical resources in Bulgarian regions. (A) Overall hospital beds (in units of people per bed); (B) ICU beds; (C) Total doctors; (D) General practitioners; (E) Cadiologists; (F) Pulmonologists; (G) Infection disease specialists; (H) Internal medicine specialists. Data from the Bulgarian National Statistical Institute (`https://www.nsi.bg/bg`)

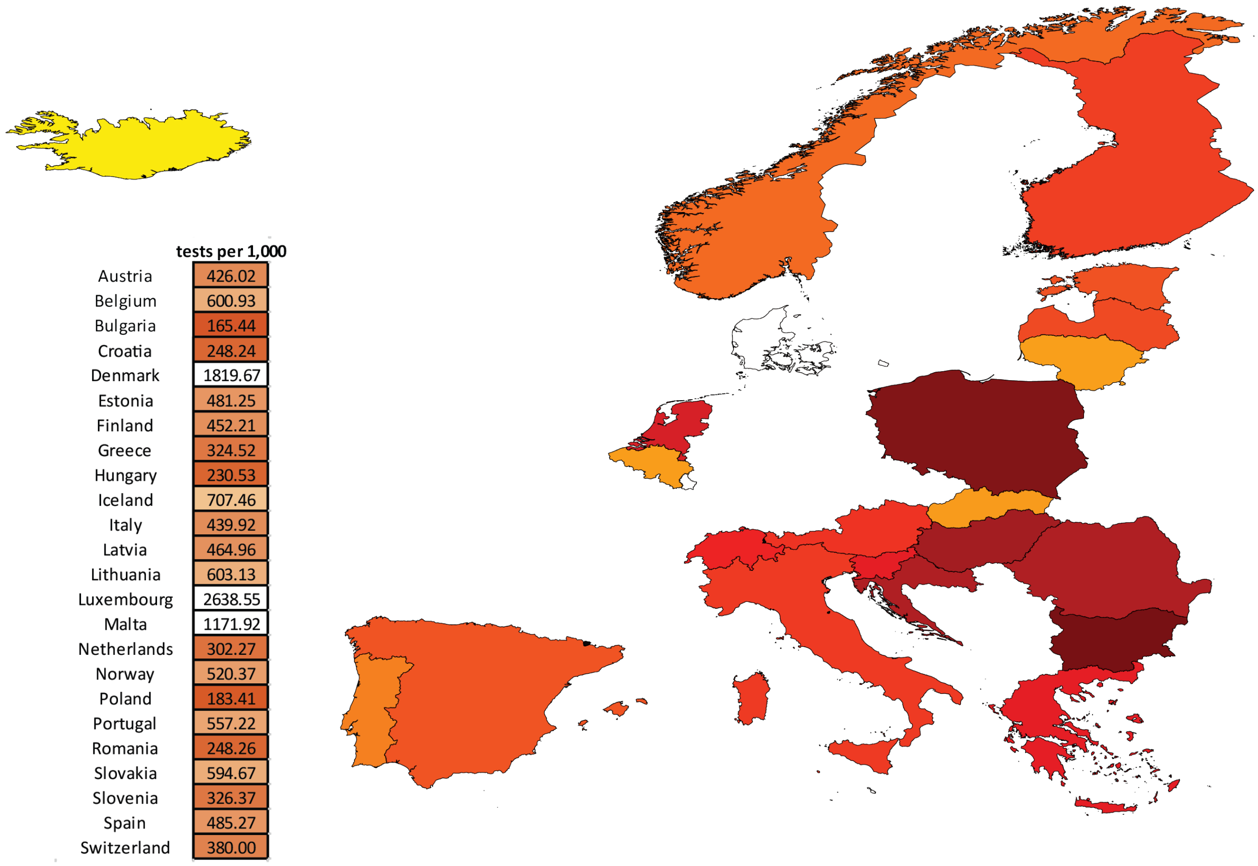

Supplementary Figure 8: COVID testing in European countries. Shown is the number of tests per 1,000 population for the period up to December 31st 2020. Data from `https://ourworldindata.org/coronavirus`

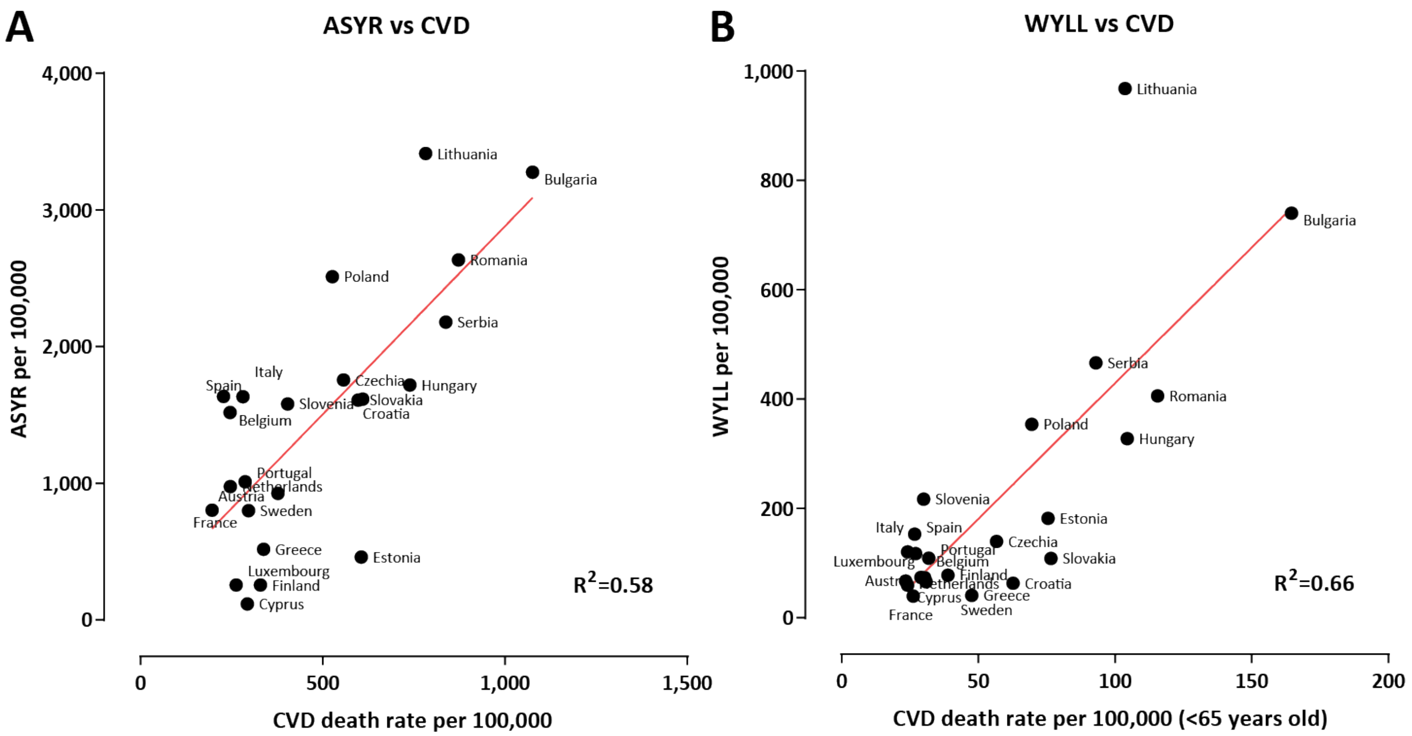

C

Correlations*	Pearson R2	Pearson P-value	Spearman r	Spearman P-value
P-Score ~ CVD	0.14775	0.49085	0.0561	0.79458
Excess Mortality ~ CVD	0.52584	0.00831	0.34522	0.0985
PYLL ~ CVD	0.60926	0.00158	0.43304	0.03454
ASYR ~ CVD	0.58387	0.00274	0.42	0.04102
WYLL ~ CVD<65	0.66024	0.00045	0.46522	0.02198

Supplementary Figure 9: Correlation between death rates due to cardiovascular disease (CVD) and ASYR and WYLL values. (A) CVD death rates per 100K population (based on Eurostat data from 2018) and ASYR values per 100K standardised population; (B) CVD death rates per 100K population (age < 65) and WYLL values per 100K working age population. (C) Correlation coefficients and p-values between CVD death rates and excess mortality metrics (p-score, EMR, PYLL, ASYR, WYLL). No adjustments were considered for the Spearman p-values in this multiple comparison because the years-of-life-lost metrics involved in three of the five hypothesis tested are closely related to each other (Spearman correlation between each pair of ASYR, WYLL and PYLL is > 0.69).
